# Expression characteristics of C-reactive protein in autoimmune diseases and their complications

**DOI:** 10.3389/fimmu.2026.1831536

**Published:** 2026-05-11

**Authors:** Qianyue Yang, Yanfang Luo, Yong Luo, Liuting Zeng, Lingyun Sun

**Affiliations:** 1 Department of Rheumatology and Immunology, Nanjing Drum Tower Hospital Clinical College of Nanjing Medical University, Nanjing, China; 2 Department of Nephrology, The Central Hospital of Shaoyang, Shaoyang, Hunan, China; 3 Department of Rheumatology and Immunology, Nanjing Drum Tower Hospital Clinical College of Nanjing University of Chinese Medicine, Nanjing, China; 4 Department of Rheumatology and Immunology, Nanjing Drum Tower Hospital, Chinese Academy of Medical Sciences & Peking Union Medical College, Nanjing, China

**Keywords:** CRP - C-reactive protein, PBC - primary biliary cholangitis, PSC - primary sclerosing cholangitis, Sjögren’s disease, SLE - systemic lupus erythematosus

## Abstract

**Background:**

Autoimmune diseases involve disruptions in immune tolerance, persistent systemic inflammation, and progressive multi-organ involvement, with increasing global prevalence. Accurate prediction of disease flares and complications, alongside tools for activity stratification, represents a significant clinical challenge. C-reactive protein (CRP), a canonical pattern recognition molecule of the pentraxin family, is a widely employed inflammatory biomarker; however, its expression patterns, cellular origins, and disease-specific roles across autoimmune conditions have not been comprehensively assessed.

**Methods:**

This study integrated retrospective clinical cohort analyses from Nanjing Drum Tower Hospital with population-level data from the National Health and Nutrition Examination Survey, proteomic profiling, and single-cell RNA sequencing to examine CRP expression across multiple autoimmune diseases, including systemic lupus erythematosus, Sjögren’s disease, autoimmune hepatitis, and various inflammatory arthritides.

**Results:**

Clinical data indicate that CRP levels exhibit considerable heterogeneity across these conditions. Proteomic analyses identify CRP as a core inflammatory mediator in conditions such as rheumatoid arthritis, while single-cell RNA sequencing delineates its major cellular sources. Integration of population-level data supports these heterogeneous patterns and demonstrates positive correlations between CRP levels and systemic inflammatory burden, as well as associations with hematologic parameters. Additionally, liver-derived single-cell and spatial transcriptomic data offer insights into the tissue-specific inflammatory landscape in autoimmune hepatitis.

**Conclusions:**

Collectively, this study maps the CRP expression landscape across multiple autoimmune diseases and identifies cellular sources in specific disease contexts. These findings indicate that CRP interpretation in autoimmune settings requires consideration of both disease type and clinical context, which may inform more refined strategies for early detection and patient stratification.

## 1 Introduction

Autoimmune diseases comprise over a hundred subtypes and are characterized by loss of immune tolerance, dysregulation of innate and adaptive immunity, chronic systemic inflammation, and progressive multi-organ damage (1). Their global incidence continues to rise, imposing significant disability, mortality, and disease burden worldwide. The spectrum of these conditions ranges from systemic connective tissue diseases including systemic lupus erythematosus (SLE), Sjögren’s disease (SjD), and rheumatoid arthritis (RA), to more organ-specific disorders such as type 1 diabetes mellitus (T1DM) and autoimmune hepatitis (AIH). Although recent advances in autoantibody detection and imaging modalities have improved diagnostic and therapeutic approaches, three major challenges persist in clinical practice. First, the insidious onset of most diseases and the paucity of specific biomarkers lead to prolonged diagnostic windows and high rates of missed diagnosis and misdiagnosis (2). Second, marked heterogeneity in inflammatory phenotypes, observed both across different diseases and among patients with the same condition, limits the utility of existing biomarkers for precise stratification of disease activity and monitoring of treatment response. Third, severe complications, including infection and organ involvement, remain the leading causes of mortality in patients with autoimmune diseases, yet reliable biomarkers for early warning and risk stratification of these complications are still lacking (3). Accordingly, the identification of inflammatory biomarkers that possess both broad-spectrum screening value and disease-specific diagnostic utility represents a critical scientific challenge that must be addressed to advance the diagnosis and management of autoimmune diseases.

C-reactive protein (CRP) is a core pattern recognition molecule of the pentraxin family within the innate immune system. It was the first acute-phase protein to be identified and remains the most widely used clinically worldwide, nearly a century after its initial discovery (4). Under physiological conditions, CRP is primarily synthesized and secreted by the liver in response to pro-inflammatory cytokine signals such as IL-6. Upon acute inflammatory stimulation, its biosynthesis can increase several hundred-fold within hours (5). Functionally, both pentameric CRP (pCRP) and monomeric CRP (mCRP) bind phosphocholine ligands in a calcium-dependent manner, thereby activating the classical complement pathway (6). This activity implicates CRP in pathogen clearance, phagocytosis of apoptotic cells, and regulation of inflammatory homeostasis, positioning it as a key molecule bridging innate immune defense and inflammatory pathology (6, 7). Notably, elevated circulating CRP levels have been identified as a well-established risk factor for cardiovascular disease, with robust epidemiological evidence supporting this association^1^.

As early as the 1980s, Pepys and colleagues first observed an atypical expression pattern of CRP in autoimmune diseases. They noted that patients with systemic lupus erythematosus (SLE) exhibit only mild to moderate elevations in serum CRP levels, even during active disease accompanied by significant IL-6 elevation and systemic inflammatory burden (8). Gabay et al. further verified this atypical expression pattern and explored its potential mechanism in a classical study [PMID: 8336307], which found no correlation between interleukin 6 (IL-6) and CRP blood levels in SLE by detecting serum IL-6 and CRP levels in 37 SLE patients and 22 rheumatoid arthritis (RA) patients, in sharp contrast to the positive correlation between the two molecules in RA; notably, the study also confirmed significantly higher IL-6 levels in SLE than in RA, suggesting an impaired acute phase response to IL-6 in SLE that underpins the abnormal CRP expression (6). This so-called “CRP paradox” has remained a central scientific question in the field (4, 5). Subsequent research has progressively elucidated the multifactorial regulation of CRP expression in SLE. Type I interferon gene signatures can significantly inhibit CRP transcriptional activation, while CRP gene polymorphisms (e.g., rs1205, rs2794521, rs3091244) directly influence basal expression levels and the capacity for inflammatory response (6, 9–12). Concurrently, the conformational conversion of pentameric CRP (pCRP) to monomeric CRP (mCRP) not only alters its ligand recognition and complement activation functions but also renders it a critical target antigen in autoimmune responses. This conversion is closely linked to SLE disease phenotypes, activity, and complications such as lupus nephritis (13–16). Furthermore, anti-CRP autoantibodies have been successively identified in various autoimmune diseases, including systemic lupus erythematosus (SLE) and systemic sclerosis (SSc, 17). Specifically, Bell SA et al. demonstrated that IgG antibodies to CRP are present in SSc patients, targeting non-native epitopes on modified CRP molecules (17). These antibodies may participate in disease pathogenesis by influencing the immunoregulatory functions of CRP, further expanding the biological significance of CRP in autoimmunity (17–19).

With advancing research, the diagnostic and therapeutic value of CRP across different autoimmune diseases is being progressively elucidated. In rheumatoid arthritis (RA), CRP is a core indicator for assessing disease activity, monitoring treatment response, and determining prognosis, and it has been integrated into the EULAR/ACR-recommended criteria for RA risk stratification and remission assessment (20, 21). In conditions such as Sjögren’s disease, autoimmune hepatitis, and systemic sclerosis, CRP levels have also been demonstrated to correlate significantly with disease activity, the extent of organ involvement, and long-term prognosis (17, 19). However, despite numerous exploratory studies on single diseases, critical scientific gaps and clinical bottlenecks persist in CRP research within the autoimmune field, severely hindering its transition from a broad-spectrum, non-specific inflammatory marker to a tool for precision diagnostics. First, previous studies have largely been limited to small cohort analyses of single diseases, lacking pan-disease, panoramic omics-level evidence. The common expression characteristics of CRP across multiple autoimmune diseases have not been systematically elucidated, and its immunological basis as a broad-spectrum inflammatory marker in autoimmunity remains incompletely defined. Second, the heterogeneity of CRP expression among different autoimmune diseases has not been systematically resolved. While distinct patterns of CRP expression and its correlation with disease activity are evident across diseases, the underlying regulatory mechanisms and disease-specific diagnostic value remain underexplored. Third, existing research has predominantly focused on CRP expression changes within the primary disease itself, with insufficient investigation into its dynamic changes, early warning potential, and diagnostic discriminatory power in the context of autoimmune disease complications. Notably, the expression pattern of CRP in non-infectious organ involvement in SLE has not been systematically characterized (22, 23). Fourth, previous studies have almost exclusively focused on circulating CRP of hepatic origin, lacking single-cell resolution analysis of the cellular sources and regulatory mechanisms of CRP synthesis within the local microenvironments of autoimmune diseases. This significantly limits a deeper understanding of CRP’s biological functions in autoimmunity. Fifth, most studies lack validation in large-scale population cohorts. The screening efficacy, diagnostic thresholds, and clinical applicability of high-sensitivity CRP (hs-CRP) in diverse autoimmune patient populations have yet to be systematically confirmed (7, 24, 25).

To address the aforementioned knowledge gaps, this study integrated a single-center retrospective clinical cohort, population-based data from the National Health and Nutrition Examination Survey (NHANES 2017-2018), comprehensive proteomic profiling, and single-cell transcriptomic sequencing to systematically investigate the expression patterns and clinical significance of C-reactive protein (CRP) across multiple autoimmune diseases from a multidimensional perspective. First, we performed comprehensive proteomic analysis focused on arthritic and vasculitic diseases to characterize CRP expression and associated inflammatory pathways at the molecular level, identifying shared regulatory networks in which CRP acts as a core inflammatory mediator. Next, we extended the investigation to a single-center clinical cohort including diverse autoimmune conditions (systemic lupus erythematosus [SLE] and Sjögren’s disease [SJD]), systematically evaluating correlations between CRP levels, disease activity, systemic inflammatory burden, and hematologic parameters. This analysis revealed inter-disease heterogeneity in CRP expression and provided cross-disease clinical validation of our proteomic findings. Concurrently, we used NHANES population data (focused on arthritis-related diseases) to examine associations between CRP, inflammatory burden, and hematologic parameters, thereby providing independent population-based confirmation of arthritis-related observations from the clinical cohort. Furthermore, using single-cell transcriptomic and spatial transcriptomic technologies, we first established a single-cell atlas from healthy individuals, followed by single-cell analysis of diseased liver tissue using autoimmune hepatitis as a model. This approach allowed us to investigate the potential cellular sources of CRP and the regulatory dynamics of the tissue microenvironment at cellular and spatial resolution, yielding mechanistic insights into CRP’s biological functions in organ-specific autoimmune contexts.

Collectively, these analyses define the CRP expression landscape across multiple autoimmune diseases through four integrated dimensions: molecular pathways (arthritis proteomics), clinical heterogeneity (multi-disease cohort), population-based validation (NHANES arthritis), and cellular-spatial architecture (liver single-cell and spatial transcriptomics). This work provides multidimensional, complementary data to advance understanding of the disease-specific roles of this canonical inflammatory molecule.

## 2 Materials and methods

### 2.1 Single-center retrospective clinical study cohort

Two independent, non-overlapping autoimmune disease sub-cohorts were constructed for this single-center study, with all participants recruited from inpatient and outpatient populations at Nanjing Drum Tower Hospital between January 2009 and December 2025, and a uniform set of exclusion criteria applied to both cohorts.

#### 2.1.1 Inclusion criteria

##### 2.1.1.1 Systemic lupus erythematosus sub-cohort

Patients enrolled in this sub-cohort were under stable conventional or targeted immunomodulatory therapy for at least 3 months, including hydroxychloroquine (HCQ), glucocorticoids, tacrolimus (TAC), mycophenolate mofetil (MMF), total glucosides of paeony (TGP), tripterygium glycosides (GTW), and cyclophosphamide (CYC). No recent dose adjustments, treatment intensification, or new induction therapies had been implemented within 3 months prior to sample collection.

Given the limited number of newly diagnosed, treatment_naïve SLE patients at our center, we included clinically stable patients on maintenance regimens to ensure an adequate and representative sample size. This strategy minimized confounding from acute therapeutic changes and allowed for reliable evaluation of CRP profiles in relation to SLE disease activity and organ involvement under real_world clinical management.

##### 2.1.1.2 Sjögren’s disease sub-cohort

Patients with pSS (including those with concomitant interstitial lung disease, ILD) diagnosed between January 2024 and December 2025 were enrolled. Participants with the following criteria are enrolled in this study: ① Age ≥ 18 years; ② Fulfillment of the 2016 ACR/EULAR classification criteria for Sjögren’s disease; ③ Complete baseline clinical data available, including CRP measurements at enrollment. All CRP samples were collected at baseline, prior to the initiation of any targeted pharmacotherapy (e.g., glucocorticoids, immunosuppressants, biologics) or induction therapy for pSS, to eliminate potential interference from therapeutic interventions.

#### 2.1.2 Exclusion criteria

##### 2.1.2.1 Systemic lupus erythematosus (SLE) sub-cohort

Participants meeting any of the following criteria were excluded from the SLE sub-cohort: ① History of acute bacterial/viral infection, trauma, or major surgery within 4 weeks pre-enrollment that may confound inflammatory biomarker measurement; ② Concurrent active malignancy, severe organ insufficiency, decompensated heart failure, or other severe systemic diseases; ③ Overlap syndrome or ≥2 concurrent autoimmune diseases; ④ Unstable immunomodulatory therapy within 3 months pre-enrollment, including significant dose adjustments, treatment intensification, or newly initiated glucocorticoids, immunosuppressants, or biologic agents. Stable maintenance doses of hydroxychloroquine (HCQ), glucocorticoids (prednisone-equivalent ≤10 mg/day), tacrolimus (TAC), mycophenolate mofetil (MMF), tripterygium glycosides (GTW), cyclophosphamide (CYC), and other conventional immunosuppressants were permitted. This criterion was strictly implemented to ensure that baseline CRP levels reflected the stable inflammatory status of SLE under routine clinical management rather than acute fluctuations caused by recent therapeutic modifications.

##### 2.1.2.2 Sjögren’s disease (SjD) sub-cohort

Participants meeting any of the following criteria were excluded from the SjD sub-cohort: ① History of acute bacterial/viral infection, trauma, or major surgery within 4 weeks pre-enrollment that may confound inflammatory biomarker measurement; ② Concurrent active malignancy, severe organ insufficiency, decompensated heart failure, or other severe systemic diseases; ③ Overlap syndrome or ≥2 concurrent autoimmune diseases; ④ Glucocorticoid exposure (prednisone-equivalent dose >10 mg/day), immunosuppressants, or biologic agents within 3 months pre-enrollment, precluding accurate assessment of disease-intrinsic inflammatory activity. This criterion was strictly implemented to ensure that baseline CRP levels reflected the intrinsic inflammatory activity of primary Sjögren’s disease independent of therapeutic interference.

#### 2.1.3 Diagnostic definitions of hepatic involvement

For the classification of hepatic abnormalities in the SLE cohort, strict distinctions were made between SLE-associated hepatic involvement (lupus hepatitis) and idiopathic autoimmune hepatitis (AIH) in accordance with published clinical consensus (26).

(1) SLE-associated hepatic involvement (lupus hepatitis) is defined as patients with a confirmed SLE diagnosis (meeting 1997 ACR or 2019 EULAR/ACR criteria) presenting with liver function abnormalities (serum alanine aminotransferase/ALT > 40 U/L, aspartate aminotransferase/AST > 40 U/L, or total bilirubin > 17.1 μmol/L) that were closely associated with SLE disease activity (SLEDAI-2000 score ≥ 6), with no evidence of AIH-specific autoantibodies (anti-smooth muscle antibody/SMA, anti-liver/kidney microsome type 1 antibody/anti-LKM1) or typical AIH liver histology (interface hepatitis, portal plasma cell infiltration), and exclusion of other causes of liver injury (viral hepatitis, drug-induced liver injury, alcoholic liver disease, etc.).

(2) Autoimmune hepatitis (AIH): Diagnosed in accordance with the 2019 EASL clinical practice guidelines for AIH, requiring the presence of AIH-specific autoantibodies, characteristic liver histopathological changes, elevated serum transaminases, and exclusion of other chronic liver diseases. Patients with overlapping SLE and AIH were excluded from the present study in accordance with the exclusion criteria in 2.1.2.

### 2.2 Public pan-disease blood proteomic dataset

The pan-proteomic data used in this study were derived from a public pan-disease high-throughput serum proteomic dataset generated on the SomaScan platform. Based on high-throughput aptamer-based detection technology, the SomaScan platform enables highly specific, sensitive, and accurate quantification of thousands of human proteins, and is widely recognized as one of the gold-standard platforms for large-sample clinical proteomic research to date.

The dataset included in this study covers serum SomaScan proteomic quantification data from pediatric healthy controls, adult healthy controls, and patients with 7 autoimmune diseases: multiple sclerosis, myositis, psoriasis, psoriatic arthritis, rheumatoid arthritis (RA), systemic sclerosis (SSc), and vasculitis. A total of 2356 samples across 32 independent cohorts were enrolled, including 1020 male samples across 27 cohorts and 1328 female samples across 30 cohorts. The detection rate of all target proteins was 100%, with 0% of samples below the limit of detection (LOD). All datasets had undergone standardized and rigorous quality control procedures in the original studies, with definitive clinical diagnoses, complete demographic data, and intact clinical covariate information for all enrolled samples, which fully supported the subsequent analyses in this study.

### 2.3 Public single-cell and spatial transcriptomic datasets

#### 2.3.1 Single-cell and spatial transcriptomic dataset of primary sclerosing cholangitis liver tissues (GSE245620)

The data for the analysis of the liver immune microenvironment and CRP expression in PSC in this study were obtained from the publicly available dataset GSE245620 in the Gene Expression Omnibus (GEO) database. This dataset contains scRNA-seq data of 47,156 cells and single-nucleus RNA sequencing (snRNA-seq) data of 23,000 nuclei from liver tissues of 10 PSC patients, 3 primary biliary cholangitis (PBC) patients, and 24 healthy donors. It is also matched with 10x Visium spatial transcriptomic data and Nanostring GeoMx Digital Spatial Profiling data, fully covering the information of the liver cellular ecosystem, spatial distribution characteristics, and regional inflammatory microenvironment under healthy and disease states.

#### 2.3.2 Single-cell transcriptomic datasets of PBC liver tissues

To further systematically dissect the expression pattern of CRP in autoimmune biliary liver diseases, we additionally included two independent public single-cell transcriptomic datasets of PBC liver tissues for integrated validation:

##### 2.3.2.1 GSE125188

This dataset contains scRNA-seq data of liver tissues from 5 PBC patients and 3 healthy donors, capturing a total of 18,240 high-quality cells that fully cover parenchymal and non-parenchymal cell subsets in the liver.

##### 2.3.2.2 GSE115469

This dataset contains scRNA-seq data of liver tissues from 8 PBC patients and 10 healthy donors, capturing a total of 102,951 high-quality cells, and is one of the largest single-cell transcriptomic datasets of PBC liver tissues available to date.

Both datasets underwent rigorous quality control in the original studies, and the clinical diagnosis of all included samples met the diagnostic criteria for PBC formulated by the European Association for the Study of the Liver (EASL). These datasets were used to compare and analyze the cellular source, expression differences of CRP, and its association with disease progression in PBC and healthy liver tissues, which, together with the PSC and AIH datasets, formed a complete analytical system for autoimmune liver diseases.

### 2.4 NHANES large-scale population validation cohort

The population validation data of this study were derived from the NHANES 2009–2020 Pre-Pandemic Data (data from 2017 to 2018), officially released by the National Center for Health Statistics (NCHS) of the Centers for Disease Control and Prevention (CDC), United States. The data are publicly and freely accessible via the official CDC website (https://wwwn.cdc.gov/nchs/nhanes/). All data analyses in this study were performed in strict accordance with the *Guidelines for High Quality Analyses of NHANES Data* issued by NCHS. The selection of the pre-pandemic study cycle can minimize the confounding interference of COVID-19 infection on systemic inflammatory biomarkers and the disease course of autoimmune diseases to the greatest extent.

#### 2.4.1 Inclusion and exclusion criteria

Participants meeting the following criteria were included in this study: ① Adult participants aged ≥ 18 years; ② Participants with complete demographic data, laboratory measurements of high-sensitivity C-reactive protein (hs-CRP), physician-confirmed arthritis diagnosis, and detailed arthritis subtype information. Participants meeting any of the following criteria were excluded from this study: ① Pregnant female participants; ② Participants with missing core diagnostic data for rheumatoid arthritis (RA), osteoarthritis (OA), or psoriatic arthritis (PsA).

#### 2.4.2 Variable extraction and grouping

The following core variables were extracted from the NHANES database: ① Demographic characteristics: age, sex, race/ethnicity, educational level, and poverty-income ratio (PIR); ② Lifestyle and clinical covariates: body mass index (BMI), smoking history, alcohol consumption history, occupational physical activity, and comorbidities including hypertension, diabetes mellitus, and dyslipidemia; ③ Laboratory test data: serum levels of high-sensitivity C-reactive protein (hs-CRP) and erythrocyte sedimentation rate (ESR); ④ Disease diagnosis information: physician-confirmed diagnosis of rheumatoid arthritis (RA), osteoarthritis (OA), and psoriatic arthritis (PsA), based on self-reported physician-confirmed results from standardized questionnaires.

The study population was divided into the case group and the healthy control group according to arthritis diagnosis status. The case group was further stratified into the rheumatoid arthritis (RA) subgroup, osteoarthritis (OA) subgroup, and psoriatic arthritis (PsA) subgroup; the healthy control group included participants without any form of arthritis or autoimmune diseases.

### 2.5 Software, reference databases and statistical analysis

#### 2.5.1 Analysis software and reference databases

Basic data processing and bioinformatic analysis were performed using the following software: Cell Ranger (v7.1.0; 10x Genomics, Pleasanton, CA, USA), SpaceRanger (v2.0.0; 10x Genomics, Pleasanton, CA, USA), R (v4.3.1; R Foundation for Statistical Computing, Vienna, Austria), SPSS (v26.0; IBM Corp., Armonk, NY, USA), and GraphPad Prism (v9.5.1; GraphPad Software, San Diego, CA, USA).

Core R packages implemented for specialized data analysis included: Seurat (v4.3.0), harmony (v0.1.1), limma (v3.56.2), ggplot2 (v3.4.4), pheatmap (v1.0.12), survey (v4.2-1), scater (v1.28.4), SingleCellExperiment (v1.22.0), SpatialExperiment (v1.10.0), DoubletFinder (v2.0.3), and rms (v6.7-0).

The following reference databases were used for sequence alignment, cell type annotation, and functional enrichment analysis: UniProt Human Reference Proteome Database (version: UP000005640), Human GRCh38 Reference Genome (version: GRCh38.p13), CellMarker Database (v2.0), PanglaoDB Database (v2.0), Gene Ontology (GO) Database, and Kyoto Encyclopedia of Genes and Genomes (KEGG) Database.

#### 2.5.2 Statistical analysis

All statistical analyses were performed in R (v4.3.1, http://www.R-project.org), with data visualization using GraphPad Prism (v9.5.1) and the ggplot2 package (v3.4.4). Normality of continuous variables was assessed via the Shapiro-Wilk test; normally distributed data were presented as mean ± standard deviation (SD), and non-normally distributed data as median (interquartile range, IQR; P25-P75). For two-group comparisons of continuous variables, independent samples t-test (normal data) or Mann-Whitney U test (non-normal data) was used; for multi-group comparisons, one-way ANOVA with Bonferroni-corrected *post hoc* tests (normal data) or Kruskal-Wallis H test with Dunn-Bonferroni-corrected *post hoc* tests (non-normal data) was applied. Pearson or Spearman rank correlation analysis was performed for bivariate data with normal or non-normal distribution, respectively, with correlations visualized via correlation plots. Benjamini-Hochberg correction was used for multiple testing in high-throughput omics differential analyses, with a false discovery rate (FDR) < 5% set as the significance threshold. All tests were two-sided, and P < 0.05 was considered statistically significant.

## 3 Results

### 3.1 Circulating CRP expression profiling in autoimmune diseases via pan-disease proteomic analysis

To characterize the circulating expression pattern of CRP across a broad spectrum of autoimmune diseases, we performed a large-scale proteomic analysis of 2356 serum samples from 32 independent clinical cohorts. All CRP measurements were above the limit of detection (LOD), ensuring the robustness and reliability of our quantitative analysis. The inclusion of healthy child controls was based on the original design of the public SomaScan dataset to maintain the integrity of the dataset and avoid selection bias; meanwhile, it provided a developmental baseline reference for CRP expression, considering the age-related differences in the basal expression of this acute-phase protein due to innate immune system maturation.

**Figure 1:**
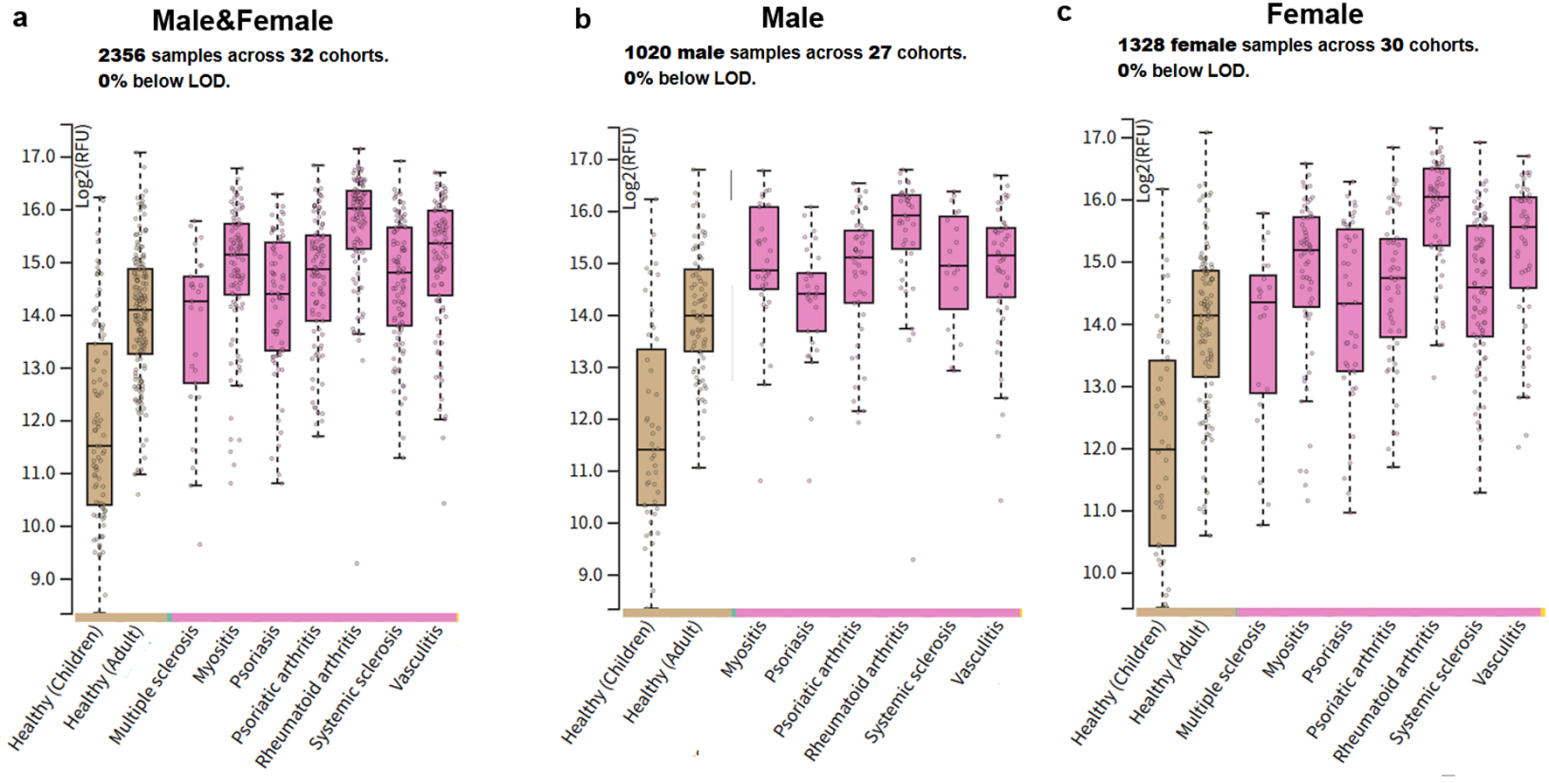
Circulating CRP expression profiles across autoimmune diseases and healthy controls (children and adults) in a pan-disease plasma proteomic dataset. **(a)** CRP expression distribution in the combined male and female cohort (2356 samples across 32 cohorts). **(b)** CRP expression distribution in the male-only sub-cohort (1020 samples across 27 cohorts). **(c)** CRP expression distribution in the female-only sub-cohort (1328 samples across 30 cohorts). Plasma CRP levels were quantified using the SomaScan assay, with expression values presented as log₂-transformed relative fluorescence units (log₂(RFU)). No CRP measurements were below the limit of detection (LOD) across all samples. Study groups includehealthy child controls (served as a developmental baseline for the physiological expression of CRP), healthy adult controls (the core baseline for adult autoimmune disease comparison), multiple sclerosis, idiopathic inflammatory myositis, psoriasis, psoriatic arthritis, rheumatoid arthritis, systemic sclerosis, and vasculitis. Box plots show the median (central line), interquartile range (box), and 1.5×IQR range (whiskers), with individual sample values overlaid as scatter points. Healthy children showed lower basal CRP expression than healthy adults, which is consistent with the physiological characteristics of pediatric innate immune system and liver function maturation.

In the combined male and female cohort (**Figure 1a**), circulating CRP levels were significantly dysregulated across all the study groups (global P < 0.001, Kruskal-Wallis test). Notably, the basal log₂(RFU) of CRP in healthy children was lower than that in healthy adults, which is consistent with the physiological characteristics of immature hepatic synthetic function and low basal expression of acute-phase proteins in the pediatric population, verifying the detection reliability of the proteomic dataset. Compared with healthy adult controls, patients with rheumatoid arthritis, systemic sclerosis, and vasculitis exhibited the most robust elevation of circulating CRP, with a 2–4 fold increase in raw relative fluorescence unit (RFU) values, corresponding to a 1–2 log₂ unit elevation in median log₂(RFU), reflecting a severe systemic inflammatory burden in these disorders. Moderate but statistically significant upregulation of CRP was also observed in patients with psoriatic arthritis, myositis, and psoriasis. In contrast, patients with multiple sclerosis showed only a mild elevation of circulating CRP, highlighting the substantial heterogeneity of systemic inflammatory phenotypes across distinct autoimmune diseases.

Given the well-documented sex dimorphism in autoimmune disease prevalence, pathogenesis, and inflammatory responses, we further stratified our analysis by biological sex. In the male sub-cohort (1020 samples from 27 cohorts, **Figure 1b**), healthy adult males had a lower baseline circulating CRP level (median log₂(RFU) ≈14.0) than healthy adult females. Consistent with the overall cohort, male patients with rheumatoid arthritis, systemic sclerosis, and vasculitis showed marked CRP upregulation, with a particularly prominent elevation observed in male patients with psoriatic arthritis. In the female sub-cohort (1328 samples from 30 cohorts, **Figure 1c**), CRP upregulation in autoimmune diseases was highly concordant with the overall cohort, with significant elevation detected in most disease groups. Notably, female patients with multiple sclerosis exhibited a greater magnitude of CRP upregulation than male patients with the same disease, suggesting a potential sex-specific regulatory effect on CRP-mediated inflammatory responses in this disorder.

Collectively, our pan-disease proteomic analysis demonstrates that circulating CRP is universally upregulated across a wide range of autoimmune diseases, with the magnitude of elevation closely correlated with the degree of systemic inflammation in each disease. Furthermore, we identified significant sex-specific differences in both baseline CRP expression and disease-associated CRP upregulation, which may provide novel insights into the sex dimorphism of autoimmune disease pathogenesis and clinical outcomes.

### 3.2 Expression characteristics of CRP in SLE and its complications

#### 3.2.1 Baseline clinical characteristics of the study population

A total of 4,534 patients with systemic lupus erythematosus (SLE) were included in this study. Among them, 1,539 patients presented with lupus nephritis (LN), comprising 33.94% of the study population, whereas 2,995 patients had no renal involvement, representing 66.06%. Detailed demographic characteristics, comorbidity distributions, and immunosuppressive medication profiles of the study population are presented in **Table 1**.

**Table 1:** Demographic characteristics of SLE cohort.

Variables	Total (n = 4534)	SLE (n = 2995)	LN (n = 1539)	Statistic	P
CRP, M (Q₁, Q₃)	4.10 (2.00, 12.20)	4.20 (1.90, 13.35)	4.10 (2.20, 9.95)	Z=-0.45	0.654
ESR, M (Q₁, Q₃)	39.00 (19.00, 70.00)	38.00 (18.00, 67.00)	43.00 (23.00, 74.00)	Z=-4.06	<.001
Age, M (Q₁, Q₃)	36.00 (26.00, 48.00)	36.00 (26.00, 49.00)	35.00 (26.00, 47.00)	Z=-3.29	<.001
BMI, M (Q₁, Q₃)	21.57 (19.61, 24.28)	21.79 (19.68, 24.27)	21.34 (19.53, 24.32)	Z=-1.06	0.290
Gender, n (%)				χ²=8.48	0.004
Male	465 (10.26)	279 (9.32)	186 (12.09)		
Female	4069 (89.74)	2716 (90.68)	1353 (87.91)		
Diabetes mellitus (DM), n (%)				χ²=4.42	0.036
No	4150 (91.53)	2760 (92.15)	1390 (90.32)		
Yes	384 (8.47)	235 (7.85)	149 (9.68)		
Hypertension (HTN), n (%)				χ²=502.30	<.001
No	3040 (67.05)	2344 (78.26)	696 (45.22)		
Yes	1494 (32.95)	651 (21.74)	843 (54.78)		
Thrombotic diseases, n (%)				χ²=42.77	<.001
No	1936 (42.70)	1382 (46.14)	554 (36.00)		
Yes	2598 (57.30)	1613 (53.86)	985 (64.00)		
Hepatic impairment, n (%)				χ²=11.07	<.001
No	3675 (81.05)	2386 (79.67)	1289 (83.76)		
Yes	859 (18.95)	609 (20.33)	250 (16.24)		
Renal impairment, n (%)				χ²=86.34	<.001
No	3939 (86.88)	2702 (90.22)	1237 (80.38)		
Yes	595 (13.12)	293 (9.78)	302 (19.62)		
Hydroxychloroquine (HCQ) usage, n (%)				χ²=29.51	<.001
No	1760 (38.82)	1247 (41.64)	513 (33.33)		
Yes	2774 (61.18)	1748 (58.36)	1026 (66.67)		
Glucocorticoid usage, n (%)				χ²=87.38	<.001
No	922 (20.34)	729 (24.34)	193 (12.54)		
Yes	3612 (79.66)	2266 (75.66)	1346 (87.46)		
Tacrolimus (TAC) usage, n (%)				χ²=64.10	<.001
No	4214 (92.94)	2849 (95.13)	1365 (88.69)		
Yes	320 (7.06)	146 (4.87)	174 (11.31)		
Mycophenolate mofetil (MMF) usage, n (%)				χ²=163.10	<.001
No	3956 (87.25)	2749 (91.79)	1207 (78.43)		
Yes	578 (12.75)	246 (8.21)	332 (21.57)		
Total glucosides of paeony (TGP) usage, n (%)				χ²=3.12	0.077
No	4377 (96.54)	2881 (96.19)	1496 (97.21)		
Yes	157 (3.46)	114 (3.81)	43 (2.79)		
Tripterygium glycosides (GTW) usage, n (%)				χ²=7.73	0.005
No	4308 (95.02)	2865 (95.66)	1443 (93.76)		
Yes	226 (4.98)	130 (4.34)	96 (6.24)		
Cyclophosphamide (CYC) usage, n (%)				χ²=107.63	<.001
No	3424 (75.52)	2404 (80.27)	1020 (66.28)		
Yes	1110 (24.48)	591 (19.73)	519 (33.72)		
SLEDAI, M (Q₁, Q₃)	10.00 (5.00, 17.00)	8.00 (4.00, 14.00)	14.00 (8.00, 20.00)	Z=-18.90	<.001
24h urine protein, M (Q₁, Q₃)	757.00 (230.00, 2402.00)	349.50 (156.00, 1067.50)	1973.00 (812.00, 4729.00)	Z=-25.30	<.001
dsDNA, M (Q₁, Q₃)	28.40 (2.70, 301.00)	32.90 (5.40, 309.50)	16.80 (1.50, 81.30)	Z=-1.51	0.132
C3, M (Q₁, Q₃)	0.69 (0.47, 0.92)	0.72 (0.50, 0.96)	0.62 (0.42, 0.84)	Z=-9.21	<.001
C4, M (Q₁, Q₃)	0.13 (0.08, 0.20)	0.14 (0.08, 0.20)	0.12 (0.07, 0.19)	Z=-4.42	<.001
IgA, M (Q₁, Q₃)	2.32 (1.68, 3.25)	2.41 (1.75, 3.35)	2.15 (1.56, 3.06)	Z=-6.49	<.001
IgE, M (Q₁, Q₃)	86.00 (40.00, 226.00)	88.00 (42.00, 230.50)	82.00 (38.00, 215.00)	Z=-1.68	0.092
IgG, M (Q₁, Q₃)	13.80 (10.00, 18.70)	14.80 (11.30, 19.70)	11.60 (8.00, 16.12)	Z=-14.83	<.001
IgM, M (Q₁, Q₃)	0.96 (0.62, 1.42)	1.03 (0.68, 1.51)	0.85 (0.54, 1.24)	Z=-9.53	<.001
WBC count, M (Q₁, Q₃)	5.20 (3.50, 7.60)	5.20 (3.40, 7.60)	5.30 (3.60, 7.50)	Z=-1.06	0.287
Neutrophil count, M (Q₁, Q₃)	3.60 (2.20, 5.80)	3.50 (2.20, 5.80)	3.70 (2.30, 5.70)	Z=-2.24	0.025
Lymphocyte pct, M (Q₁, Q₃)	20.00 (12.90, 28.80)	20.60 (13.40, 29.20)	18.85 (12.30, 27.60)	Z=-3.69	<.001
Hemoglobin, M (Q₁, Q₃)	107.00 (89.00, 121.00)	110.00 (94.00, 124.00)	100.00 (82.00, 116.00)	Z=-11.83	<.001
Platelet, M (Q₁, Q₃)	160.00 (101.00, 217.00)	160.00 (101.00, 219.00)	159.00 (100.00, 215.00)	Z=-0.47	0.637
Cholesterol, M (Q₁, Q₃)	4.29 (3.51, 5.25)	4.06 (3.40, 4.91)	4.83 (3.89, 6.04)	Z=-15.89	<.001
Triglycerides, M (Q₁, Q₃)	1.59 (1.09, 2.34)	1.47 (1.01, 2.13)	1.85 (1.26, 2.76)	Z=-12.38	<.001
HDL, M (Q₁, Q₃)	1.03 (0.77, 1.35)	1.02 (0.75, 1.34)	1.05 (0.79, 1.36)	Z=-2.52	0.012
LDL, M (Q₁, Q₃)	2.23 (1.69, 2.90)	2.10 (1.62, 2.65)	2.56 (1.93, 3.50)	Z=-15.00	<.001
Albumin, M (Q₁, Q₃)	33.60 (28.90, 37.50)	35.00 (31.20, 38.30)	30.40 (25.40, 34.85)	Z=-21.14	<.001
TBil, M (Q₁, Q₃)	6.90 (5.00, 10.20)	7.60 (5.50, 10.90)	5.70 (4.20, 8.30)	Z=-16.10	<.001
ALT, M (Q₁, Q₃)	18.10 (11.90, 32.40)	19.50 (12.50, 34.30)	16.20 (10.85, 27.55)	Z=-8.08	<.001
AST, M (Q₁, Q₃)	21.80 (16.10, 33.50)	22.70 (16.80, 35.60)	20.10 (15.00, 30.40)	Z=-7.47	<.001
Creatinine, M (Q₁, Q₃)	53.00 (43.00, 73.00)	50.00 (41.00, 62.00)	65.00 (48.00, 118.50)	Z=-19.74	<.001
Urea, M (Q₁, Q₃)	5.40 (4.00, 8.10)	4.90 (3.70, 6.60)	7.10 (4.80, 12.33)	Z=-20.54	<.001
eGFR, M (Q₁, Q₃)	125.20 (87.60, 159.40)	135.55 (105.38, 165.72)	99.70 (52.70, 142.00)	Z=-14.79	<.001
VitD, M (Q₁, Q₃)	13.19 (8.71, 19.09)	15.13 (10.52, 21.07)	10.23 (6.46, 15.79)	Z=-8.46	<.001

SLE, Systemic Lupus Erythematosus; LN, Lupus Nephritis; HCQ, Hydroxychloroquine; TAC, Tacrolimus; MMF, Mycophenolate Mofetil; TGP, Total Glucosides of Paeony; GTW, Tripterygium Glycosides; CYC, Cyclophosphamide; χ², Chi-square test.

In terms of gender distribution, females comprised 89.74% of the total population. Notably, the proportion of male patients was significantly higher in the LN group compared with SLE patients without renal involvement (12.09% vs. 9.32%; χ² = 8.48, P = 0.004). Analysis of comorbidities indicated that the LN group exhibited significantly higher prevalence rates of diabetes mellitus, hypertension, thrombotic diseases, and renal insufficiency relative to SLE patients without renal involvement, with all intergroup differences achieving statistical significance (all P < 0.05). These comorbidities, combined with elevated CRP levels—a known risk factor for cardiovascular disease^1^—further increase the cardiovascular risk burden in LN patients. In contrast, the prevalence of liver dysfunction was significantly lower in the LN group than in SLE patients without renal involvement (16.24% vs. 20.33%; χ² = 11.07, P < 0.001). The liver dysfunction statistics in this cohort exclusively referred to SLE-associated hepatic involvement (lupus hepatitis) as defined in Section 2.1.3, with all cases of overlapping AIH excluded prior to cohort enrollment.

Regarding medication usage, the utilization rates of hydroxychloroquine, glucocorticoids, tacrolimus (TAC), mycophenolate mofetil (MMF), Tripterygium glycosides (GTW), and cyclophosphamide (CYC) were all significantly higher in the LN group compared with SLE patients without renal involvement, with statistically significant differences observed between the groups (all P < 0.05). No statistically significant difference was observed in the usage rate of total glucosides of paeony (TGP) between the two groups (χ² = 3.12, P = 0.077).

#### 3.2.2 Differential expression of CRP, inflammatory biomarkers and disease activity in SLE patients with and without lupus nephritis

**Figure 2:**
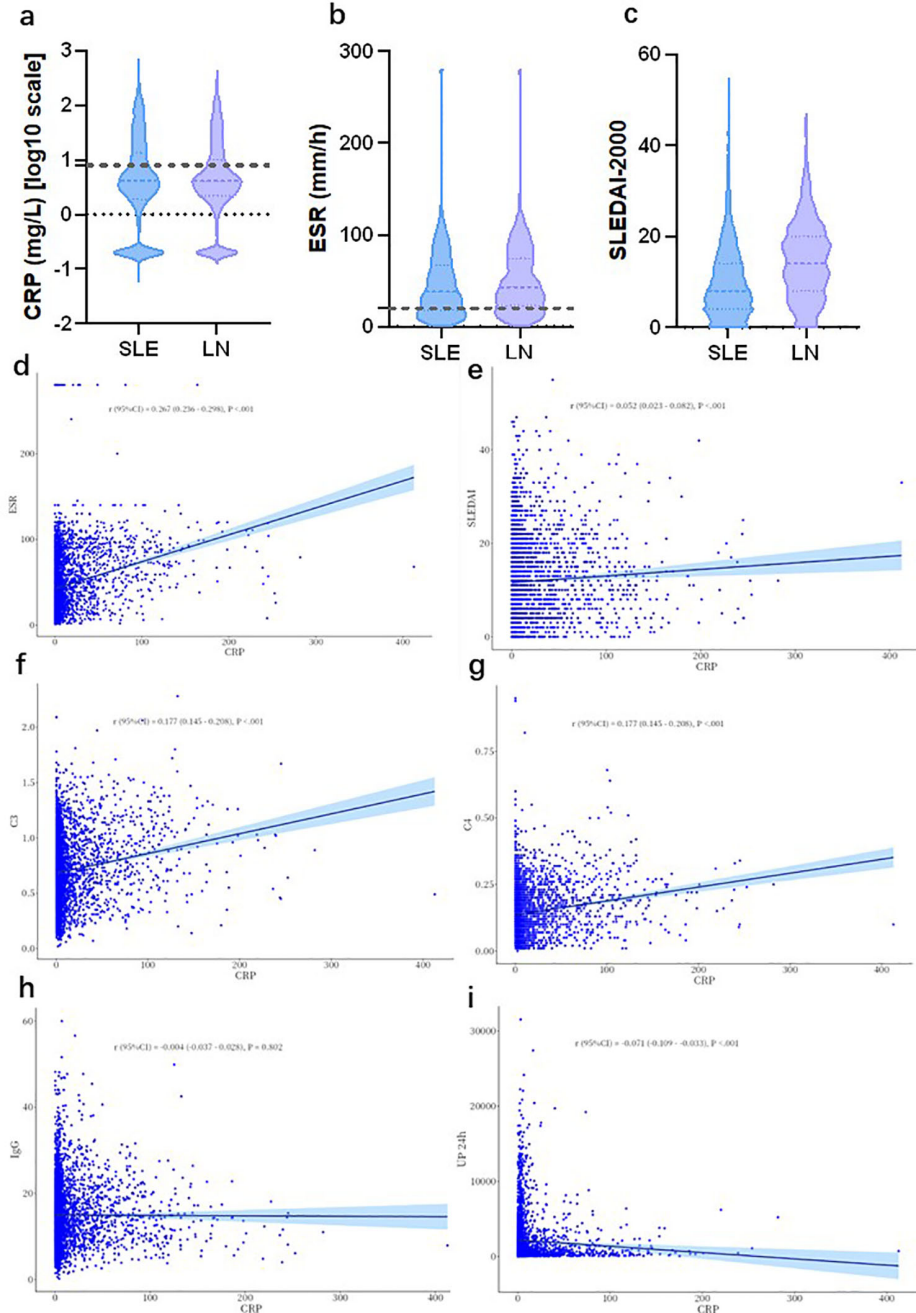
Circulating CRP expression and its clinical correlations in SLE patients with and without LN. **(a-c)** Violin plots comparing **(a)** log10-transformed CRP levels, **(b)** ESR, and **(c)** SLEDAI-2000 scores between SLE patients without renal involvement and LN patients (Mann-Whitney U test). d-i: Spearman rank correlation analyses between circulating CRP levels and **(d)** ESR, **(e)** SLEDAI-2000, **(f)** C3, **(g)** C4, **(h)** IgG, and **(i)** 24-hour urinary protein in the overall SLE cohort. Scatter plots with fitted regression lines and 95%CIs are shown, with correlation coefficients, 95%CIs and *P* values annotated in each panel. CRP, C-reactive protein; SLE, systemic lupus erythematosus; LN, lupus nephritis; ESR, erythrocyte sedimentation rate; SLEDAI-2000, Systemic Lupus Erythematosus Disease Activity Index 2000; CI, confidence interval; C3, complement 3; C4, complement 4; IgG, immunoglobulin G.

A total of 4274 SLE patients with complete CRP measurement data were enrolled in this analysis, including 1471 patients (34.42%) with LN and 2803 patients (65.58%) with SLE without renal involvement. The median CRP level of the total cohort was 4.10 mg/L (interquartile range [IQR]: 2.00–12.20 mg/L). Specifically, the median CRP level was 4.20 mg/L (IQR: 1.90–13.35 mg/L) in SLE patients without renal involvement, and 4.10 mg/L (IQR: 2.20–9.95 mg/L) in patients with LN. Mann-Whitney U test revealed no statistically significant difference in circulating CRP levels between the two groups (*Z* = -0.45, *P* = 0.654), and the distribution of CRP levels in the two groups is presented in **Figure 2a**.

For the analysis of erythrocyte sedimentation rate (ESR), a total of 3610 patients with complete ESR measurement data were included, among whom 1225 (33.93%) were diagnosed with LN and 2385 (66.07%) had SLE without renal involvement. The median ESR level of the total cohort was 39.00 mm/h (IQR: 19.00–70.00 mm/h). Patients in the LN group had significantly higher ESR levels than those in the SLE without renal involvement group (43.00 mm/h vs. 38.00 mm/h, *Z* = -4.06, *P* < 0.001), with the distribution characteristics between groups shown in **Figure 2b**. Meanwhile, violin plot analysis demonstrated that the Systemic Lupus Erythematosus Disease Activity Index 2000 (SLEDAI-2000) score was significantly higher in the LN group than in the SLE without renal involvement group, with a statistically significant between-group difference (**Figure 2c**).

Notably, despite higher disease activity and a greater burden of systemic comorbidities in patients with LN, circulating CRP levels did not differ significantly from those in SLE patients without renal involvement (26). Although CRP is a classic marker of systemic inflammation—encoded on chromosome 1p and primarily driven by IL-6 (with synergistic enhancement by IL-1β) via STAT3, C/EBP, and NF-κB transcription factors (27)—its synthesis is uniquely suppressed in active SLE. This dissociation arises because type I interferon (IFN-I), the core driver of SLE pathogenesis, inhibits hepatic CRP transcription through two distinct mechanisms: first, IFN-I activates STAT1/STAT2 via the JAK-STAT pathway, competitively suppressing the STAT3 activation required for CRP transcription; second, IFN-I upregulates the liver inhibitory protein (LIP) isoform of C/EBPβ, which antagonizes the transactivating LAP isoform essential for IL-6/IL-1β–mediated CRP induction. Consequently, even amidst significant systemic inflammation, CRP elevation is blunted in SLE, resulting in the characteristic clinical dissociation between ESR and CRP (28–30).

#### 3.2.3 Correlation analysis between circulating CRP levels and core clinical and laboratory parameters in patients with SLE

Spearman rank correlation analysis was performed to investigate the associations between circulating CRP levels and core clinical and laboratory parameters in patients with SLE, with the results presented in **Figures 2d-i**. In the overall SLE cohort, circulating CRP levels exhibited significant linear correlations with multiple clinical parameters. Specifically, CRP levels were positively correlated with ESR (*r* = 0.267, 95% CI 0.226–0.298, *P* < 0.001, **Figure 2d**) and SLEDAI-2000 scores (*r* = 0.052, 95% CI 0.023–0.082, *P* < 0.001, **Figure 2e**), suggesting that circulating CRP levels may, to a certain extent, reflect the systemic inflammatory burden and overall disease activity in patients with SLE.

In addition, circulating CRP levels were positively correlated with serum complement 3 (C3) levels (*r* = 0.177, 95% CI 0.145–0.208, *P* < 0.001, **Figure 2f**) and complement 4 (C4) levels (*r* = 0.177, 95% CI 0.145–0.208, *P* < 0.001, **Figure 2g**), and negatively correlated with 24-hour urinary protein (UP 24h) levels (*r* = -0.071, 95% CI -0.109 to -0.033, *P* < 0.001, **Figure 2i**). In contrast, no significant linear correlation was observed between circulating CRP levels and serum immunoglobulin G (IgG) concentrations (*r* = -0.004, 95% CI -0.037 to 0.028, *P* = 0.802, **Figure 2h**).

#### 3.2.4 Nonlinear dose-response relationships of circulating CRP and ESR Levels with the risk of lupus nephritis onset

**Figure 3:**
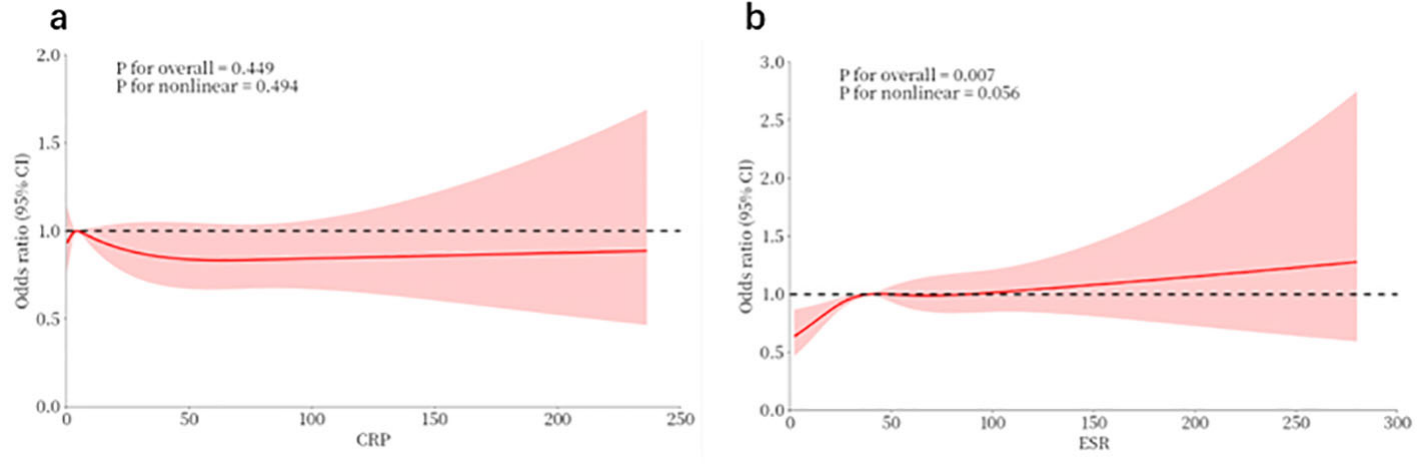
Dose-response relationships of circulating CRP and ESR with lupus nephritis onset risk in SLE patients. Restricted cubic spline models were constructed to evaluate the associations. **(a)** Association between circulating C-reactive protein (CRP) levels and lupus nephritis (LN) onset risk. **(b)** Association between erythrocyte sedimentation rate (ESR) levels and LN onset risk. The solid line indicates the odds ratio (OR), the shaded area indicates the 95% confidence interval (CI), and the dashed line marks the null effect of OR = 1. *P* values for overall and nonlinear associations are annotated in each corresponding panel. SLE, systemic lupus erythematosus; CRP, C-reactive protein; ESR, erythrocyte sedimentation rate; LN, lupus nephritis; OR, odds ratio; CI, confidence interval.

Restricted cubic spline (RCS) models were constructed to fit the associations of circulating CRP and ESR levels with the risk of lupus nephritis (LN) onset, with the results presented in **Figures 3a**, **b**, respectively.

For circulating CRP levels, the overall effect test of the model revealed no statistically significant association between circulating CRP levels and the risk of LN onset (*P* for overall = 0.449). Meanwhile, the nonlinear effect test indicated no significant nonlinear characteristic in this association (*P* for nonlinear = 0.494). As shown in the dose-response curve, with a CRP level of 0 set as the reference, the odds ratio (OR) for LN onset presented a slight downward trend with increasing circulating CRP levels. However, the 95% confidence interval (CI) of the effect size crossed the invalid line of OR = 1 throughout the entire observed range of CRP levels, which further confirmed the absence of a statistically significant association between circulating CRP levels and LN onset risk.

For ESR levels, the overall effect test of the model demonstrated a statistically significant association between ESR levels and the risk of LN onset (*P* for overall = 0.007). The nonlinear effect test showed that the association between ESR levels and LN onset risk presented a nonlinear characteristic with borderline statistical significance (*P* for nonlinear = 0.056). Specifically, with an ESR level of 0 set as the reference, the OR for LN onset was below 1 when ESR was maintained at a low level. With the elevation of ESR levels, the risk of LN onset showed a continuous upward trend, with the OR value gradually increasing above 1 and the 95% CI no longer crossing the invalid line of OR = 1, indicating that elevated ESR levels were significantly and positively correlated with an increased risk of LN onset.

### 3.3 Expression of C-reactive protein and its association with the risk of Sjögren’s disease and its complications

#### 3.3.1 Comparison of CRP levels between SjD patients and non-SjD controls

Baseline characteristics, including general clinical data, laboratory indicators, and disease-related symptoms, were compared between SjD patients and non-SjD controls (**Table 2**). The median (interquartile range, IQR) CRP level in the total study population was 2.20 (1.30, 4.30). CRP expression differed significantly between the two groups (Z = -10.98, P < 0.001), with a higher median (IQR) level in SjD patients [3.10 (1.90, 6.50)] than in non-SjD controls [1.40 (1.00, 3.00)]. Regarding other inflammatory indicators, erythrocyte sedimentation rate (ESR), CRP-to-albumin ratio (CAR), and CRP-to-lymphocyte ratio (CLR) were significantly elevated in SjD patients compared with non-SjD controls (all P < 0.001), whereas no significant difference was observed in interleukin-6 (IL-6) levels between the two groups (Z = -1.02, P = 0.306). These results indicate that CRP is more sensitive than IL-6 in reflecting systemic inflammation in SjD, and that combining CRP with albumin or lymphocytes to form CAR and CLR may better characterize the inflammatory profile of SjD patients. Among disease-related symptoms, the incidence of dry eye (χ² = 3.89, P = 0.049) and dry mouth (χ² = 4.01, P = 0.045) was significantly different between SjD patients and non-SjD controls, representing typical clinical manifestations of SjD. Furthermore, the positive rates of anti-nuclear antibody (ANA), anti-Ro52 antibody, anti-SSA antibody, and anti-SSB antibody were significantly higher in SjD patients than in non-SjD controls (all P < 0.001), which further supports the diagnostic classification of SjD patients in this study.

**Table 2:** Comparison of demographic characteristics, clinical symptoms, laboratory parameters, and inflammatory indicators between SjD patients and non-SjD controls.

Variables	Total (n = 749)	Non-SjD controls (n = 368)	SjD patients (n = 381)	Statistic	P
C Reactive Protein, M (Q₁, Q₃)	2.20 (1.30, 4.30)	1.40 (1.00, 3.00)	3.10 (1.90, 6.50)	Z=-10.98	<.001
Erythrocyte Sedimentation Rate, M (Q₁, Q₃)	12.00 (7.00, 20.00)	8.00 (6.00, 13.00)	16.00 (8.00, 30.00)	Z=-9.29	<.001
Interleukin 6, M (Q₁, Q₃)	5.15 (2.10, 15.88)	4.35 (2.68, 5.53)	5.33 (2.12, 17.41)	Z=-1.02	0.306
CAR, M (Q₁, Q₃)	0.05 (0.03, 0.09)	0.03 (0.02, 0.06)	0.06 (0.04, 0.12)	Z=-7.97	<.001
CLR, M (Q₁, Q₃)	1.77 (0.82, 3.83)	1.18 (0.63, 2.75)	2.30 (1.12, 4.82)	Z=-6.81	<.001
Gender, n(%)				χ²=0.88	0.349
Male	61 (8.75)	35 (9.72)	26 (7.72)		
Female	636 (91.25)	325 (90.28)	311 (92.28)		
Age, Mean ± SD	52.93 ± 14.10	52.81 ± 13.96	53.05 ± 14.24	t=-0.23	0.816
Smoking, n(%)				χ²=0.62	0.432
No	418 (96.98)	211 (96.35)	207 (97.64)		
Yes	13 (3.02)	8 (3.65)	5 (2.36)		
Drinking, n(%)				χ²=0.00	1
No	427 (99.07)	217 (99.09)	210 (99.06)		
Yes	4 (0.93)	2 (0.91)	2 (0.94)		
HTN, n(%)				χ²=3.31	0.069
No	315 (84.00)	123 (88.49)	192 (81.36)		
Yes	60 (16.00)	16 (11.51)	44 (18.64)		
DM, n(%)				χ²=0.39	0.530
No	337 (90.11)	127 (91.37)	210 (89.36)		
Yes	37 (9.89)	12 (8.63)	25 (10.64)		
BMI, M (Q₁, Q₃)	23.10 (20.50, 25.08)	22.85 (20.48, 25.00)	23.40 (22.33, 25.78)	Z=-1.40	0.162
Symptom duration, M (Q₁, Q₃)	2.00 (1.00, 4.00)	2.00 (1.00, 4.00)	2.00 (1.00, 5.00)	Z=-1.33	0.183
Dry eye, n(%)				χ²=3.89	0.049
No	177 (28.46)	112 (31.55)	65 (24.34)		
Yes	445 (71.54)	243 (68.45)	202 (75.66)		
Dry mouth, n(%)				χ²=4.01	0.045
No	268 (43.37)	140 (39.89)	128 (47.94)		
Yes	350 (56.63)	211 (60.11)	139 (52.06)		
Anti-Nuclear Antibody, n(%)				χ²=150.78	<.001
Negative	142 (50.35)	120 (88.24)	22 (15.07)		
Positive	140 (49.65)	16 (11.76)	124 (84.93)		
Anti-Ro52 Antibody, n(%)				–	<.001
Negative	185 (65.14)	129 (96.99)	56 (37.09)		
Positive	95 (33.45)	4 (3.01)	91 (60.26)		
Weak Positive	4 (1.41)	0 (0.00)	4 (2.65)		
Anti-SSA Antibody, n(%)				–	<.001
Negative	179 (63.93)	130 (97.74)	49 (33.33)		
Positive	97 (34.64)	2 (1.50)	95 (64.63)		
Weak Positive	4 (1.43)	1 (0.75)	3 (2.04)		
Anti-SSB Antibody, n(%)				–	<.001
Negative	241 (85.77)	133 (100.00)	108 (72.97)		
Positive	35 (12.46)	0 (0.00)	35 (23.65)		
Weak Positive	5 (1.78)	0 (0.00)	5 (3.38)		
Hemoglobin, M (Q₁, Q₃)	123.00 (112.00, 133.00)	120.00 (107.00, 132.00)	125.00 (116.00, 134.00)	Z=-4.18	<.001
Lymphocyte Count, M (Q₁, Q₃)	1.50 (1.00, 1.90)	1.60 (0.80, 1.90)	1.40 (1.10, 1.90)	Z=-1.81	0.070
Neutrophil Count, M (Q₁, Q₃)	3.30 (2.60, 4.70)	3.50 (2.85, 4.80)	3.10 (2.30, 4.50)	Z=-3.95	<.001
Platelet Count, M (Q₁, Q₃)	212.00 (175.00, 230.00)	219.00 (201.00, 227.75)	193.00 (143.00, 233.50)	Z=-5.79	<.001
White Blood Cell Count, M (Q₁, Q₃)	5.60 (4.60, 6.90)	5.80 (4.90, 6.80)	5.40 (4.30, 7.10)	Z=-1.76	0.078
Complement C3, M (Q₁, Q₃)	1.12 (0.97, 1.27)	1.17 (1.06, 1.32)	1.11 (0.95, 1.25)	Z=-2.49	0.013
Complement C4, M (Q₁, Q₃)	0.22 (0.15, 0.27)	0.25 (0.19, 0.33)	0.22 (0.14, 0.27)	Z=-2.98	0.003
Immunoglobulin A, M (Q₁, Q₃)	2.44 (1.72, 3.59)	2.41 (1.61, 2.94)	2.44 (1.73, 3.66)	Z=-0.34	0.732
Immunoglobulin E, M (Q₁, Q₃)	22.00 (9.00, 93.00)	18.00 (8.20, 81.50)	22.50 (11.00, 94.50)	Z=-0.78	0.433
Immunoglobulin G, M (Q₁, Q₃)	13.10 (10.90, 16.80)	11.35 (9.55, 12.65)	14.10 (11.50, 17.45)	Z=-5.59	<.001
Immunoglobulin M, M (Q₁, Q₃)	1.13 (0.84, 1.54)	1.05 (0.90, 1.37)	1.14 (0.83, 1.58)	Z=-0.17	0.868
Albumin, M (Q₁, Q₃)	43.90 (41.52, 45.60)	44.70 (43.60, 46.70)	42.60 (39.62, 44.70)	Z=-9.03	<.001
Alanine Aminotransferase, M (Q₁, Q₃)	20.60 (14.90, 30.30)	24.75 (17.00, 33.45)	17.30 (12.80, 25.40)	Z=-5.85	<.001
Aspartate Aminotransferase, M (Q₁, Q₃)	24.60 (20.10, 28.90)	26.00 (22.33, 29.08)	21.70 (17.55, 28.10)	Z=-5.72	<.001
Total Bilirubin, M (Q₁, Q₃)	9.45 (7.18, 12.43)	11.20 (9.05, 13.80)	9.20 (7.00, 12.10)	Z=-2.82	0.005
Glutathione Reductase, M (Q₁, Q₃)	67.00 (48.00, 72.50)	58.50 (45.25, 68.00)	67.00 (49.00, 73.00)	Z=-0.91	0.362
Creatinine, M (Q₁, Q₃)	54.00 (47.00, 64.00)	64.40 (52.00, 75.70)	53.00 (46.70, 61.60)	Z=-3.27	0.001
Cystatin C, M (Q₁, Q₃)	0.89 (0.71, 1.05)	0.56 (0.51, 0.58)	0.94 (0.77, 1.10)	Z=-3.19	0.001
Urea, M (Q₁, Q₃)	5.00 (3.90, 6.05)	4.90 (3.68, 5.33)	5.00 (4.00, 6.10)	Z=-0.83	0.409
eGFR, M (Q₁, Q₃)	115.60 (95.50, 135.85)	115.90 (99.00, 135.88)	115.60 (95.00, 135.70)	Z=-0.18	0.855
Urine Occult Blood, n(%)				χ²=21.94	<.001
Negative	187 (78.90)	77 (96.25)	110 (70.06)		
Positive	26 (10.97)	1 (1.25)	25 (15.92)		
Weak Positive	24 (10.13)	2 (2.50)	22 (14.01)		
Urine Protein, n(%)				χ²=25.06	<.001
Negative	194 (82.91)	80 (100.00)	114 (74.03)		
Positive	11 (4.70)	0 (0.00)	11 (7.14)		
Weak Positive	29 (12.39)	0 (0.00)	29 (18.83)		

SjD, Sjögren’s disease; CRP, C-reactive protein; CAR, CRP-to-albumin ratio; CLR, CRP-to-lymphocyte ratio; HTN, hypertension; DM, diabetes mellitus; BMI, body mass index; eGFR, estimated glomerular filtration rate; SD, standard deviation; M, median; Q₁, 1st quartile; Q₃, 3rd quartile; t, t-test; Z, Mann-Whitney test; χ², Chi-square test.

#### 3.3.2 Logistic regression analysis of CRP and SjD risk

To clarify the association between CRP level and the risk of SjD, a logistic regression analysis was performed with SjD as the dependent variable and CRP (divided by quartile) as the independent variable, adjusted by different confounding factors. This analysis was the focus of the present result, aiming to determine whether CRP could serve as an independent risk factor for SjD. The logistic regression results showed that the association between CRP level and SjD risk was significant and dose-dependent. In the unadjusted model, compared with the Q1 group (reference group), the risk of SjD in the Q3 and Q4 groups of CRP was significantly increased, with odds ratios (OR) of 4.4 (95% confidence interval [CI]: 2.9, 6.9) and 7.8 (95% CI: 4.9, 12.3), respectively (both P<.001). After adjusting for gender and age (Model I), the significant association remained unchanged: the OR values for Q3 and Q4 groups were 4.3 (95% CI: 2.7, 6.9) and 8.1 (95% CI: 5.0, 13.1), respectively (both P<.001). After further adjusting for smoking status and drinking status (Model II), the risk of SjD was further increased with the increase of CRP level, and the OR values of Q3 and Q4 groups were 11.4 (95% CI: 5.5, 23.9) and 25.4 (95% CI: 12.1, 53.3), respectively (both P<.001). These results confirmed that CRP was an independent risk factor for SjD, and the risk of SjD increased with the elevation of CRP level. In addition, logistic regression analysis of CAR and CLR (divided by quartile) also showed similar results (**Table 3**), further confirming that CRP-related inflammatory ratios were closely associated with the occurrence of SjD.

**Table 3:** Association of CRP, CAR, and CLR quartiles with SjD risk across different adjustment models.

Exposure	Group	Non-adjusted	Adjust I model	Adjust II model
C-reactive protein, quartile	Q1	1.0 (Ref.)	1.0 (Ref.)	1.0 (Ref.)
	Q2	1.3 (0.8, 2.0) 0.228	1.3 (0.8, 2.0) 0.293	1.9 (0.9, 4.0) 0.093
	Q3	4.4 (2.9, 6.9) <0.001	4.3 (2.7, 6.9) <0.001	11.4 (5.5, 23.9) <0.001
	Q4	7.8 (4.9, 12.3) <0.001	8.1 (5.0, 13.1) <0.001	25.4 (12.1, 53.3) <0.001
Sample size		749	697	412
CAR, quartile	Q1	1.0 (Ref.)	1.0 (Ref.)	1.0 (Ref.)
	Q2	1.2 (0.7, 1.9) 0.595	1.2 (0.7, 2.1) 0.474	1.8 (0.6, 5.3) 0.287
	Q3	4.7 (2.7, 8.1) <0.001	4.3 (2.4, 7.8) <0.001	11.2 (4.0, 31.3) <0.001
	Q4	5.2 (3.0, 9.0) <0.001	5.8 (3.2, 10.4) <0.001	20.8 (7.4, 58.5) <0.001
Sample size		502	452	234
CLR, quartile	Q1	1.0 (Ref.)	1.0 (Ref.)	1.0 (Ref.)
	Q2	0.8 (0.4, 1.2) <0.001	0.9 (0.4, 1.3) <0.001	1.7 (0.9, 2.4) <0.001
	Q3	1.2 (0.8, 1.7) <0.001	1.2 (0.8, 1.7) <0.001	2.2 (1.4, 3.0) <0.001
	Q4	1.4 (0.9, 1.8) <0.001	1.5 (1.0, 1.9) <0.001	2.7 (2.0, 3.5) <0.001
Sample size		669	623	365

SjD, Sjögren’s disease; CRP, C-reactive protein; CAR, CRP-to-albumin ratio; CLR, CRP-to-lymphocyte ratio; Ref., reference; OR, odds ratio; CI, confidence interval. Data are presented as OR (95% CI) with P values. Model I adjusted for gender and age. Model II adjusted for gender, age, smoking status, and drinking status.

#### 3.3.3 CRP levels in SjD patients stratified by ILD and RTA status

SjD is a systemic autoimmune disease that may involve multiple organs, with ILD being a common and prognostically significant complication. We therefore further compared CRP expression levels between SjD patients with and without ILD (**Table 4**). Among the 381 SjD patients included, 277 were classified as SjD without ILD and 104 as SjD-ILD. The median (IQR) CRP level in SjD-ILD patients was 3.90 (2.18, 7.33), which was higher than that in SjD patients without ILD [2.90 (1.80, 6.30)]; however, this difference did not reach statistical significance (Z = -1.74, P = 0.083). Notably, ESR levels were significantly elevated in SjD-ILD patients compared to those without ILD (Z = -2.04, P = 0.041), and CLR levels also showed a statistically significant difference between the two groups (Z = -2.11, P = 0.035). These findings suggest that although CRP alone did not demonstrate a statistically significant difference between the two subgroups, the observed trend toward higher CRP levels in SjD-ILD patients may reflect underlying pulmonary inflammatory injury associated with SjD. Moreover, combining CRP with ESR or CLR may offer adjunctive value in the assessment of SjD-ILD.

**Table 4:** Comparison of inflammatory indicators between SjD patients with and without ILD.

Variables	Total (n = 381)	SjD (n = 277)	SjD-ILD (n = 104)	Statistic	P
C Reactive Protein, M (Q₁, Q₃)	3.10 (1.90, 6.50)	2.90 (1.80, 6.30)	3.90 (2.18, 7.33)	Z=-1.74	0.083
CAR, M (Q₁, Q₃)	0.06 (0.04, 0.12)	0.06 (0.04, 0.13)	0.06 (0.04, 0.10)	Z=-0.67	0.502
CLR, M (Q₁, Q₃)	2.30 (1.12, 4.82)	2.18 (1.00, 4.67)	2.65 (1.64, 5.12)	Z=-2.11	0.035
Erythrocyte Sedimentation Rate, M (Q₁, Q₃)	12.00 (7.00, 18.00)	10.00 (7.00, 17.75)	13.00 (7.50, 28.50)	Z=-2.04	0.041
Interleukin 6, M (Q₁, Q₃)	7.45 (2.30, 21.63)	7.53 (3.10, 18.40)	4.14 (1.79, 26.95)	Z=-0.46	0.642

SjD, Sjögren’s disease; ILD, interstitial lung disease; CRP, C-reactive protein; CAR, CRP-to-albumin ratio; CLR, CRP-to-lymphocyte ratio; M, median; Q₁, 1st quartile; Q₃, 3rd quartile; Z, Mann-Whitney test.

We also examined CRP expression levels in SjD patients stratified by the presence of RTA, another recognized extraglandular manifestation of SjD (**Table 5**). Among the 381 SjD patients, 371 did not have RTA and 10 were diagnosed with SjD-RTA. No significant difference in CRP levels was observed between SjD-RTA patients and those without RTA (Z = -0.66, P = 0.507). Similarly, CAR, CLR, ESR, and IL-6 levels did not differ significantly between the two groups (all P > 0.05). It should be noted, however, that the sample size of the SjD-RTA group was limited to 10 cases, which may have resulted in insufficient statistical power and thus limits the reliability of these findings. Accordingly, the potential association between CRP levels and RTA in SjD warrants further investigation with expanded sample sizes.

**Table 5:** Comparison of inflammatory indicators between SjD patients with and without RTA.

Variables	Total (n = 381)	SjD (n = 371)	SjD-RTA (n = 10)	Statistic	P
C Reactive Protein, M (Q₁, Q₃)	3.10 (1.90, 6.50)	3.20 (1.95, 6.50)	2.60 (1.78, 3.63)	Z=-0.66	0.507
CAR, M (Q₁, Q₃)	0.06 (0.04, 0.12)	0.06 (0.04, 0.12)	0.05 (0.04, 0.07)	Z=-0.85	0.395
CLR, M (Q₁, Q₃)	2.30 (1.12, 4.82)	2.27 (1.12, 4.83)	2.94 (1.94, 3.09)	Z=-0.39	0.696
Erythrocyte Sedimentation Rate, M (Q₁, Q₃)	12.00 (7.00, 18.00)	12.00 (7.00, 18.00)	14.00 (8.00, 37.25)	Z=-0.76	0.450
Interleukin 6, M (Q₁, Q₃)	7.45 (2.30, 21.63)	7.45 (2.25, 21.85)	5.87 (5.00, 6.73)	Z=-0.21	0.833

SjD, Sjögren’s disease; RTA, renal tubular acidosis; CRP, C-reactive protein; CAR, CRP-to-albumin ratio; CLR, CRP-to-lymphocyte ratio; M, median; Q₁, 1st quartile; Q₃, 3rd quartile; Z, Mann-Whitney test.

In summary, CRP expression levels are significantly elevated in SjD patients compared to non-SjD controls, and logistic regression analysis confirms CRP as an independent risk factor for SjD. Although CRP did not show statistically significant differences between SjD patients with and without ILD or RTA—with the RTA comparison being constrained by small sample size—the trend toward higher CRP levels in SjD-ILD patients suggests that CRP may be associated with the severity of organ involvement in SjD. Collectively, these results indicate that CRP may serve as a potential clinical biomarker for the diagnosis and disease evaluation of SjD, and provide new insights for further mechanistic studies on the pathogenesis of SjD.

### 3.4 Expression characteristics and clinical correlations of C-reactive protein in arthritis populations based on the large-scale NHANES population cohort

The analysis in this section used the publicly available database of the 2017–2018 cycle (Cycle J) of the US National Health and Nutrition Examination Survey (NHANES). A total of 4497 eligible participants were enrolled according to pre-specified inclusion and exclusion criteria, to systematically validate the expression profiles and clinical relevance of high-sensitivity C-reactive protein (hsCRP) in populations with inflammatory arthritis including rheumatoid arthritis (RA).

#### 3.4.1 Baseline clinical characteristics of the study population

Among the 4497 enrolled participants, 1122 patients with diagnosed arthritis were included, accounting for 24.95% of the total cohort; 3375 healthy controls without a history of arthritis served as the control group, accounting for 75.05% of the total population. The 1122 arthritis patients were further stratified into four subgroups by disease type: 636 cases of osteoarthritis (OA, 56.68% of the arthritis population), 301 cases of rheumatoid arthritis (RA, 26.83%), 25 cases of psoriatic arthritis (PsA, 2.23%), and 160 cases of other types of arthritis (14.26%).

Between-group balance tests for baseline characteristics revealed a statistically significant difference in gender distribution across the arthritis subgroups (χ² = 14.80, *P* = 0.002), with the highest proportion of female patients in the OA group (64.15%) and the highest proportion of male patients in the PsA group (52.00%). A statistically significant difference was also observed in the prevalence of hypertension among the subgroups (Fisher’s exact test, *P* = 0.012), with the highest prevalence in the OA group (64.78%) and the lowest in the PsA group (40.00%). In contrast, there was no statistically significant difference in the prevalence of diabetes mellitus across the four subgroups (χ² = 9.26, *P* = 0.159), indicating favorable comparability of metabolism-related baseline characteristics among the arthritis subgroups (**Table 6**).

**Table 6:** Demographic characteristics of arthritis cohort.

Variables	Total (n = 1122)	1 (n = 636)	2 (n = 301)	3 (n = 25)	4 (n = 160)	Statistic	P
Hscrp, M (Q₁, Q₃)	2.47 (1.07, 5.34)	2.42 (1.12, 5.29)	2.37 (1.02, 4.95)	5.63 (2.18, 7.52)	2.59 (1.03, 5.01)	χ²=5.90#	0.117
Age, M (Q₁₁, p₃)	64.00 (55.00, 74.00)	66.00 (56.00, 75.00)	63.00 (55.00, 71.00)	58.00 (49.00, 67.00)	61.00 (50.75, 72.00)	χ²=18.47#	<0.001
PIR, M (Q₁, Q₃)	2.02 (1.18, 4.00)	2.20 (1.35, 4.19)	1.75 (1.00, 3.66)	1.22 (0.84, 2.53)	2.02 (1.17, 4.07)	χ²=17.63#	<0.001
Gender, n(%)						χ²=14.80	0.002
Male	457 (40.73)	228 (35.85)	140 (46.51)	13 (52.00)	76 (47.50)		
Female	665 (59.27)	408 (64.15)	161 (53.49)	12 (48.00)	84 (52.50)		
HTN, n(%)						—	0.012
Yes	695 (61.94)	412 (64.78)	179 (59.47)	10 (40.00)	94 (58.75)		
No	425 (37.88)	223 (35.06)	122 (40.53)	14 (56.00)	66 (41.25)		
Unknown	2 (0.18)	1 (0.16)	0 (0.00)	1 (4.00)	0 (0.00)		
DM, n(%)						χ²=9.26	0.159
Yes	301 (26.83)	165 (25.94)	88 (29.24)	3 (12.00)	45 (28.12)		
No	770 (68.63)	434 (68.24)	204 (67.77)	21 (84.00)	111 (69.38)		
Unknown	51 (4.55)	37 (5.82)	9 (2.99)	1 (4.00)	4 (2.50)		
Arthritis Age, M (Q₁, Q₃)	50.00 (40.00, 60.00)	50.00 (40.00, 60.00)	50.00 (39.00, 60.00)	46.00 (36.00, 55.00)	50.00 (40.00, 63.00)	χ²=4.99#	0.172
Neu, M (Q₁, Q₃)	58.60 (51.88, 64.80)	59.00 (52.30, 65.23)	56.90 (50.35, 63.50)	60.20 (53.30, 66.00)	59.90 (52.70, 65.12)	χ²=9.66#	0.022
PLT, M (Q₁, Q₃)	229.00 (195.00, 274.00)	229.00 (192.75, 277.25)	224.00 (194.75, 265.50)	254.00 (206.00, 281.00)	233.00 (200.75, 278.50)	χ²=3.52#	0.319
Hb, M (Q₁, Q₃)	13.70 (12.80, 14.70)	13.70 (12.80, 14.70)	13.70 (12.78, 14.70)	14.20 (13.00, 15.10)	13.90 (12.88, 14.70)	χ²=2.63#	0.451

#: Kruskal–Wallis test χ²: Chi-square test —: Fisher exact test M: Median Q₁: 1st Quartile Q₃: 3rd Quartile.

#### 3.4.2 Expression profiles of serum hsCRP in arthritis populations

The mean serum hsCRP level of the total study population was 4.12 ± 8.11 mg/L. Overall between-group comparison showed that patients with arthritis had significantly higher serum hsCRP levels than healthy controls without arthritis (5.36 ± 10.27 mg/L vs. 3.71 ± 7.20 mg/L), with a statistically significant difference confirmed by independent samples t-test (*t* = 4.97, *P* < 0.001).

Subgroup analysis by arthritis subtype showed that the mean hsCRP level was 5.25 ± 10.03 mg/L in the OA group, 4.70 ± 7.80 mg/L in the RA group, 7.77 ± 11.05 mg/L in the PsA group, and 6.62 ± 14.34 mg/L in the other arthritis group. One-way analysis of variance (ANOVA) revealed no statistically significant difference in hsCRP levels among the four arthritis subgroups (*F* = 1.71, *P* = 0.164). Violin plots of log10-transformed hsCRP further validated this result: although hsCRP levels in the OA, RA, PsA, and other arthritis groups were all significantly higher than those in the healthy control group, no significant difference in hsCRP distribution was observed among the arthritis subtypes (**Figure 5a**).

#### 3.4.3 Correlation analysis between serum hsCRP levels and inflammation-related hematological parameters

**Figure 4:**
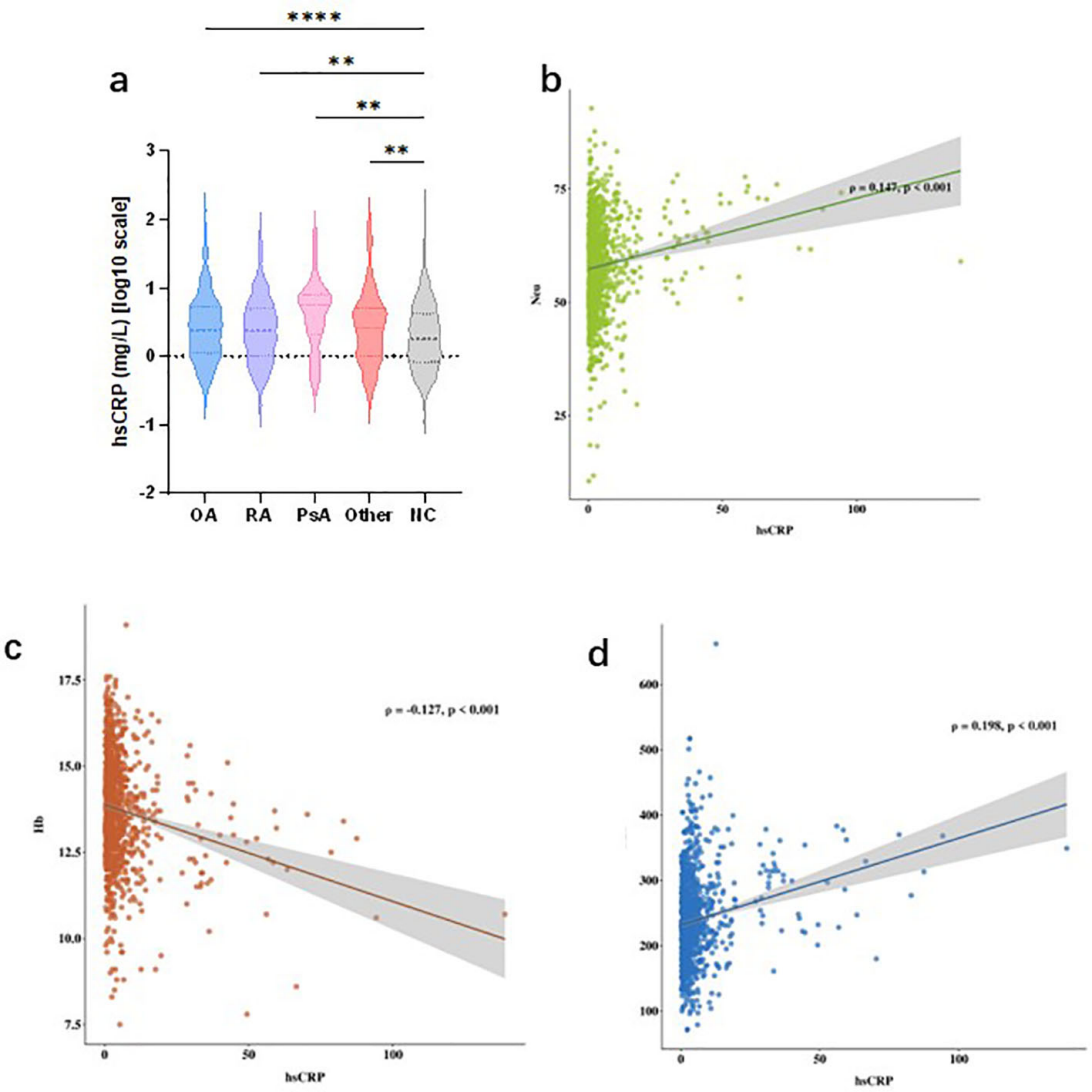
Serum hsCRP levels and their correlations with hematological inflammatory markers in the NHANES cohort. **(a)** Violin plots of log10-transformed serum hsCRP levels in patients with OA, RA, PsA, other arthritis subtypes, and healthy controls. Significance levels for between-group comparisons are annotated as *P<0.01, **P<0.001, ***P<0.0001. **(b-d)**: Scatter plots with fitted regression lines and 95% CIs showing the Pearson correlations between serum hsCRP levels and **(b)** neutrophil count, **(c)** hemoglobin level, and **(d)** platelet count in the total cohort. Correlation coefficients and P values are annotated in each panel. hsCRP, high-sensitivity C-reactive protein; NHANES, National Health and Nutrition Examination Survey; OA, osteoarthritis; RA, rheumatoid arthritis; PsA, psoriatic arthritis; CI, confidence interval.

Pearson correlation analysis was performed to explore the associations between serum hsCRP levels and core inflammation-related parameters in complete blood count, with the results presented in **Figures 4b-d**. In the total study population, serum hsCRP levels were positively correlated with peripheral blood neutrophil count (*r* = 0.147, *P* < 0.001, **Figure 4b**), negatively correlated with hemoglobin level (*r* = -0.127, *P* < 0.001, **Figure 4c**), and positively correlated with platelet count (*r* = 0.198, *P* < 0.001, **Figure 4d**). These findings indicated that circulating hsCRP levels are closely associated with systemic inflammatory burden and anemia of chronic disease status, and can effectively reflect the severity of systemic inflammation.

### 3.5 Cell-specific expression profile of CRP in healthy liver and autoimmune cholangiopathies at the single-cell transcriptomic level

To systematically delineate the cellular sources and expression patterns of CRP in the physiological liver and in the pathological states of autoimmune cholangiopathies at single-cell resolution, this study integrated multiple public single-cell transcriptomic datasets of human liver tissue. Following rigorous quality control, batch effect correction, dimensionality reduction, clustering, and cell type annotation, we characterized the distribution of CRP expression in healthy liver, primary sclerosing cholangitis (PSC), and primary biliary cholangitis (PBC).

#### 3.5.1 Cell type–specific expression profile of CRP in healthy liver

**Figure 5:**
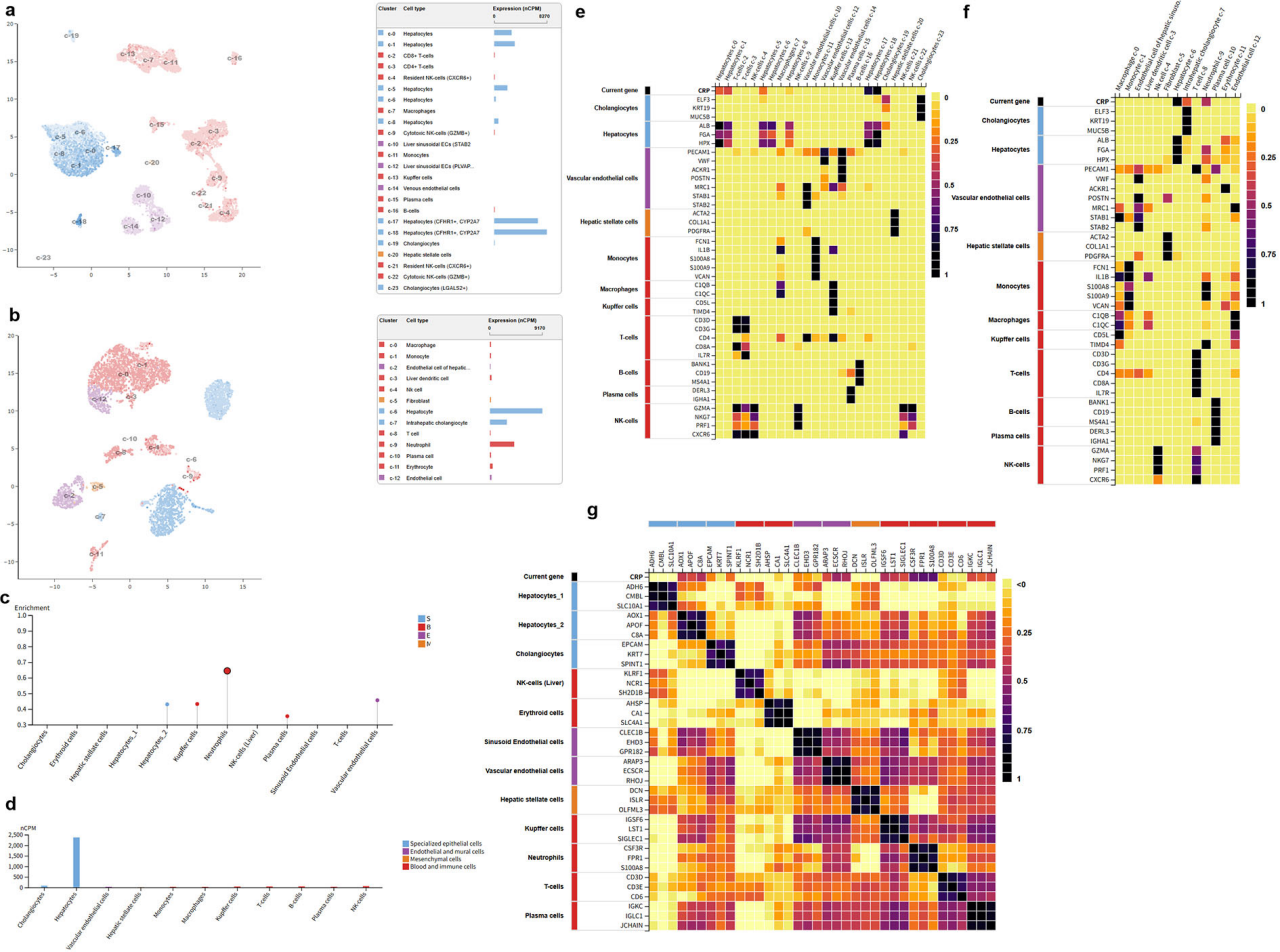
Single-cell transcriptomic landscape of CRP expression in healthy human liver. **(a)** UMAP plot of healthy liver single cells with cell type annotation, and the bar plot shows CRP expression (CPM) in each cell subpopulation. **(b)** UMAP plot of an independent healthy liver single-cell dataset, with cell type annotation and corresponding CRP expression level. **(c)** Dot plot of CRP expression enrichment in major liver cell types. **(d)** Bar plot of CRP expression across all main liver cell subpopulations. (e-g): Heatmaps of signature gene expression for cell type annotation validation in parenchymal/stromal cells **(e)**, myeloid cells **(f)**, and lymphoid cells **(g)**. UMAP, Uniform Manifold Approximation and Projection; CPM, Counts Per Million.

Based on single-cell transcriptomic data from healthy human liver, high-quality datasets were obtained after quality control. UMAP dimensionality reduction and cell type annotation identified all major cellular compartments of the liver, including hepatocytes, cholangiocytes, vascular endothelial cells, hepatic stellate cells, Kupffer cells, monocytes, macrophages, T lymphocytes, B lymphocytes, and NK cells—representing parenchymal, stromal, and immune lineages. Feature gene expression heatmaps for each subset further confirmed the accuracy of cell type annotation and clustering robustness (**Figures 5a**, **b**, **e–g**).

Expression profiling revealed that CRP expression in healthy liver is highly cell type specific. Under physiological conditions, CRP expression was predominantly enriched in the cholangiocyte subset, with the highest abundance (CPM) among all liver cell populations. Hepatocytes exhibited only minimal constitutive baseline expression. In contrast, vascular endothelial cells, hepatic stellate cells, myeloid lineages (monocytes, macrophages, Kupffer cells), and lymphocyte subsets (T, B, and NK cells) showed virtually no detectable baseline CRP expression (**Figures 5a, c, d**). These findings establish that, under physiological conditions, cholangiocytes represent the primary cellular source of CRP expression in the liver, hepatocytes serve as a secondary source, and other intrahepatic cell types do not contribute to baseline CRP expression.

#### 3.5.2 Reconstruction of CRP expression profile in the hepatic microenvironment of patients with PSC and PBC

**Figure 6:**
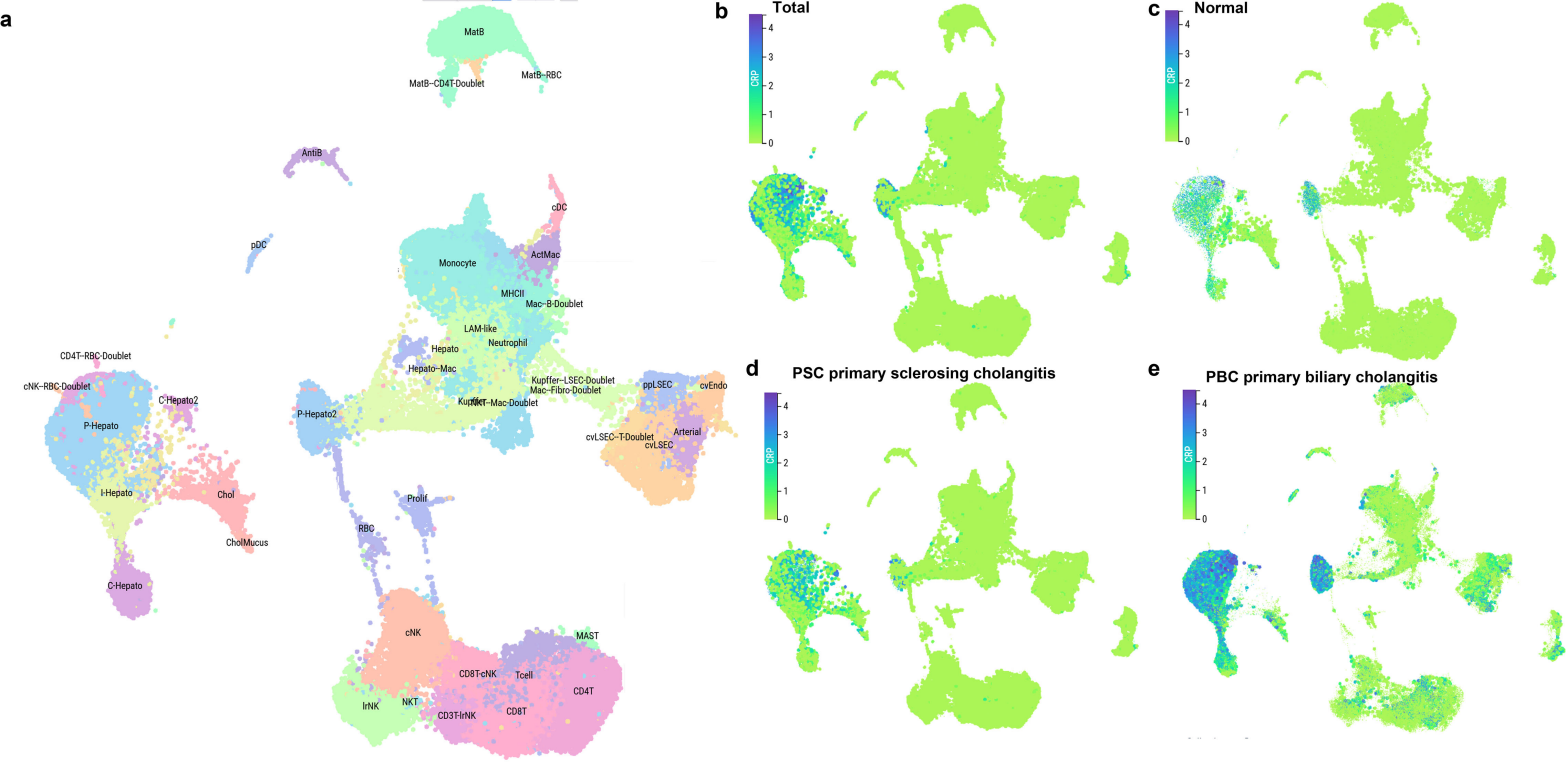
Single-cell transcriptomic profile of CRP in autoimmune hepatobiliary diseases. **(a)** Integrated UMAP plot of 189,938 high-quality liver single cells from healthy controls, PSC patients and PBC patients, with cell type annotation. **(b-e)** UMAP plots showing CRP expression distribution in the total cohort **(b)**, healthy controls **(c)**, PSC patients **(d)**, and PBC patients **(e)**. The color scale indicates normalized CRP expression level. PSC, primary sclerosing cholangitis; PBC, primary biliary cholangitis; UMAP, Uniform Manifold Approximation and Projection.

To elucidate the alterations in hepatic CRP expression under the pathological conditions of autoimmune hepatobiliary diseases, we integrated liver single-cell transcriptomic datasets from healthy controls, patients with PSC, and patients with PBC. After unified quality control and batch effect correction, a total of 189,938 high-quality hepatic single cells were obtained. Following dimensionality reduction, clustering, and cell annotation, we identified 38 cell subpopulations with well-defined biological characteristics, and constructed a high-resolution single-cell transcriptomic atlas of the liver covering both normal and disease states (**Figure 6a**). On this basis, we systematically dissected the expression and distribution patterns of CRP across different disease conditions.

In the integrated analysis of all samples, CRP expression exhibited a prominent cell-type-specific enrichment pattern, with predominant expression localized to hepatocyte and cholangiocyte subpopulations. Sparse scattered expression was detected in myeloid inflammatory cells, while the basal expression level in lymphocyte subpopulations was extremely low (**Figure 6b**). In healthy normal liver tissues, CRP maintained only an extremely low basal expression level in cholangiocyte and hepatocyte subpopulations, with no positive CRP expression detected in all other intrahepatic stromal and immune cell subpopulations. This finding was consistent with the aforementioned analytical results of healthy liver tissues, further validating the cell-type-specific expression pattern of CRP under physiological conditions (**Figure 6c**).

**Figure 7:**
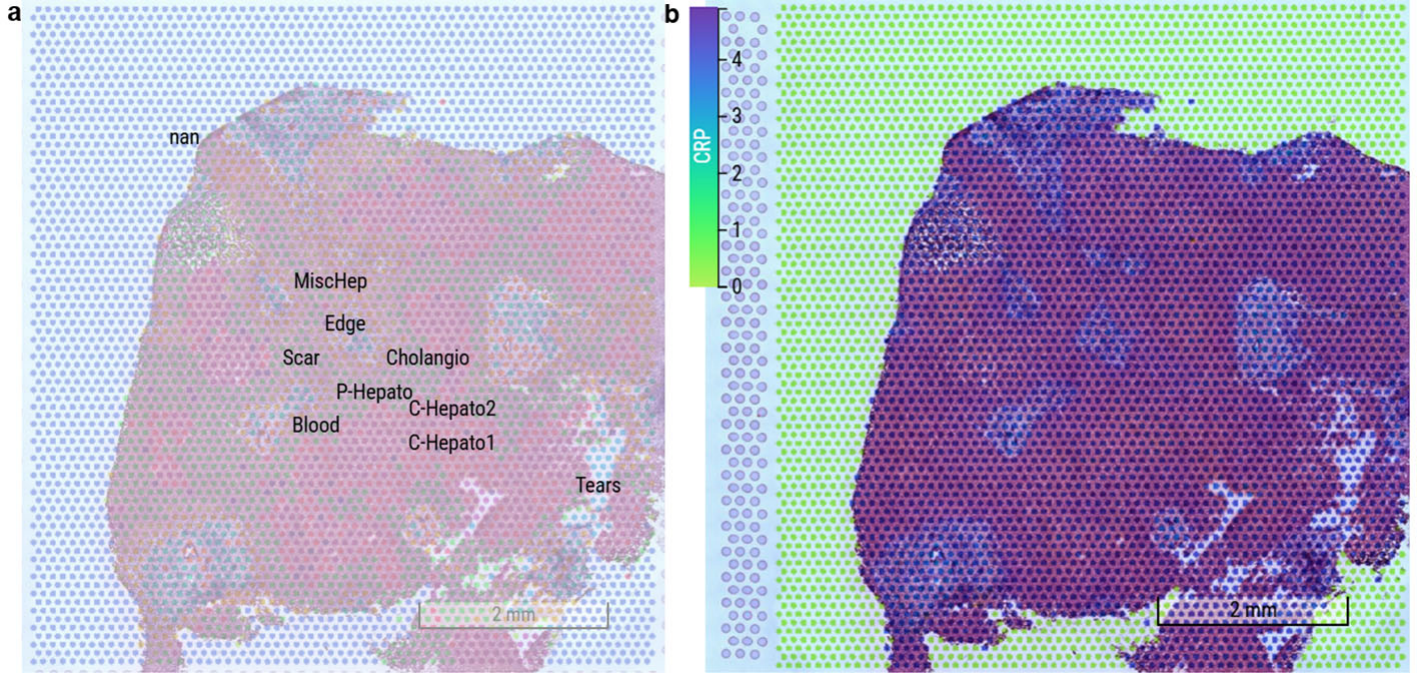
Spatial expression profile of CRP in the liver of a PSC patient. **(a)** Spatial annotation of major hepatic compartments in PSC liver tissue, including MiscHep (miscellaneous hepatocytes), Edge (edge hepatocytes), Cholangio (cholangiocytes), Scar (scar tissue), P-Hepato (pericentral hepatocytes), C-Hepato1/2 (central hepatocyte subpopulations 1 and 2), Blood (blood vessel regions), and Tears (tear artifacts). Scale bar, 2 mm. **(b)** Spatial expression map of CRP in the same PSC liver section. CRP expression is upregulated in central/pericentral hepatocytes, with ectopic signals detected in cholangiocytes, scar tissue, and blood vessel regions, reflecting broad activation of CRP expression in the fibro-inflammatory microenvironment of PSC. Color scale: 0 (low expression) to 4 (high expression) relative CRP expression. Scale bar, 2 mm.

Under the pathological state of autoimmune hepatobiliary diseases, both the expression level and expression scope of CRP in liver tissues underwent significant disease-specific remodeling. In liver tissues from patients with PSC, the overall expression level of CRP was significantly upregulated compared with that in healthy controls, with hepatocytes and biliary epithelial cells remaining the core cell types for CRP expression. Meanwhile, marked ectopic positive expression of CRP emerged in inflammation-related myeloid cell subpopulations including monocytes and activated macrophages; scattered upregulation of CRP expression was also observed in liver sinusoidal endothelial cells and infiltrating lymphocyte subpopulations. These findings suggest that the chronic inflammatory and fibrotic microenvironment in the PSC liver can induce CRP upregulation across multiple cell subpopulations (**Figure 6d**). In liver tissues from patients with PBC, the magnitude of CRP upregulation was even more pronounced. In addition to the substantial elevation of CRP expression in hepatocyte and biliary epithelial cell subpopulations, widespread positive CRP expression was detected in nearly all intrahepatic infiltrating immune cell subpopulations, including monocytes/macrophages, B lymphocytes, and T lymphocytes. Both the expression scope and expression intensity of CRP in the PBC group were significantly higher than those in the PSC group and healthy control group. These results indicate that the aberrant expression pattern of CRP is closely correlated with the disease type of autoimmune hepatobiliary diseases and the severity of local hepatic inflammation (**Figure 7e**).

#### 3.5.3 Spatial transcriptomic analysis reveals remodeled CRP expression in the PSC liver microenvironment

To further characterize the spatial distribution and cellular context of CRP expression in primary sclerosing cholangitis (PSC), we performed high-resolution spatial transcriptomic profiling on PSC patient liver tissues, alongside histological compartment annotation (**Figure 7a**).

Spatial annotation of the PSC liver section defined distinct tissue subcompartments: miscellaneous hepatocytes (MiscHep), edge hepatocytes (Edge), cholangiocytes (Cholangio), scar tissue (Scar), pericentral hepatocytes (P-Hepato), central hepatocyte subpopulations 1 and 2 (C-Hepato1/2), blood vessel regions (Blood), and tear artifacts (Tears). This spatial framework enabled precise mapping of CRP expression within the chronic inflammatory and fibrotic liver microenvironment of PSC.

Spatial expression analysis of CRP (**Figure 7b**) revealed a striking disease-specific remodeling pattern compared to healthy controls. In the PSC liver, CRP expression was broadly upregulated and spatially expanded across multiple hepatic compartments. Strong CRP signals were concentrated in the central and pericentral hepatocyte regions (C-Hepato1/2, P-Hepato), confirming parenchymal hepatocytes as a core source of CRP in PSC. Notably, ectopic CRP expression was detected in non-parenchymal compartments including cholangiocytes (Cholangio), scar tissue (Scar), and blood vessel (Blood) regions, indicating that the fibro-inflammatory microenvironment of PSC drives CRP upregulation in both parenchymal and non-parenchymal cell populations. Scattered CRP-positive signals were also observed in the MiscHep and Edge regions, further verifying the widespread activation of CRP expression in the PSC liver.

Collectively, these spatial transcriptomic data (**Figure 7**) provide direct visual evidence for the spatial expansion and ectopic activation of CRP expression in the PSC liver. While physiological CRP expression is restricted to cholangiocytes and hepatocytes at low basal levels, PSC pathology induces broad upregulation of CRP in parenchymal hepatocytes and ectopic expression in fibrotic and inflammatory compartments, co-localizing with regions of tissue injury. These findings align with our single-cell transcriptomic results, confirming that the chronic inflammatory and fibrotic microenvironment of PSC drives multi-compartmental CRP upregulation, and link local hepatic CRP production to the systemic elevation of circulating CRP observed in our clinical cohort.

## 4 Discussion

Through an integrated multi-dimensional analysis encompassing a single-center retrospective clinical cohort, pan-disease panoramic proteomics, liver single-cell transcriptomic sequencing, and nationally representative large-scale population data from NHANES, this study systematically delineated the expression landscape of CRP across ten representative autoimmune diseases and their complications, including SLE, SjD, RA, PSC, and PBC. For the first time, we comprehensively elucidated the expression patterns, immunological basis, and clinical translational value of CRP in autoimmune diseases from the perspectives of cross-disease common upregulation characteristics, inter-disease expression heterogeneity, cellular source resolution at the single-cell level, and clinical validation in large-scale populations—with proteomic profiling focusing on 7 specific autoimmune diseases including multiple sclerosis and vasculitis, clinical cohort analysis centering on SLE and SjD, and single-cell transcriptomic analysis targeting autoimmune cholangiopathies such as PSC and PBC. This study not only addresses the long-standing core scientific question over the nearly century since CRP’s discovery regarding how CRP is expressed and what biological functions it performs in autoimmune diseases, but also fills critical gaps in previous research, including the limitations of single-disease small cohort analyses, lack of pan-disease panoramic proteomic evidence, insufficient elucidation of cellular sources in local microenvironments, and absence of validation in large representative populations. Our findings provide systematic evidence to advance the clinical translation of CRP from a broad-spectrum nonspecific inflammatory marker to a precision stratification tool for autoimmune diseases.

One of the core findings of this study is that we systematically confirmed C-reactive protein (CRP) as a shared core upregulated inflammatory molecule across multiple autoimmune diseases via pan-disease panoramic proteomics on the SomaScan platform, providing a panoramic, precise interpretation and supplement to the long-standing “CRP paradox” in the field of autoimmunity. The classical view holds that a “CRP paradox” exists in IFN-I-driven autoimmune diseases represented by systemic lupus erythematosus (SLE): even during active disease phase, patients only present with mild to moderate elevation of serum CRP, which is mismatched with the pathological features of elevated systemic inflammatory burden and interleukin-6 (IL-6) levels. Early studies even suggested that CRP has no significant correlation with disease activity of SLE and Sjögren’s Disease (SJD (31, 32). However, our large-scale proteomic analysis based on 2356 samples from 32 independent clinical cohorts showed that circulating CRP levels presented consistent and universal upregulation across all 7 included autoimmune diseases, with only significant inter-disease heterogeneity in the magnitude of upregulation. Specifically, the most prominent CRP elevation was observed in patients with rheumatoid arthritis (RA), systemic sclerosis (SSc), and vasculitis, which are dominantly driven by the IL-6 inflammatory pathway; moderate upregulation was detected in patients with psoriatic arthritis (PsA), myositis, and psoriasis; while only mild upregulation was found in patients with multiple sclerosis (MS). These results directly confirmed that the magnitude of CRP upregulation is closely correlated with the activation pattern of the core inflammatory pathway of each disease. This finding does not negate previous research conclusions on the “CRP paradox”, but provides a more systematic interpretation from the pan-disease omics level: as a canonical acute-phase reactant, CRP upregulation is a shared feature of systemic inflammatory activation in autoimmune diseases. The difference in the magnitude of upregulation across diseases is essentially attributed to the disease specificity of upstream regulatory pathways. In IFN-I-driven diseases, the transcriptional activation of CRP is significantly negatively regulated by interferon signaling, resulting in a lower upregulation magnitude than in diseases centered on the IL-6/janus kinase (JAK)/signal transducer and activator of transcription 3 (STAT3) pathway. Nevertheless, the core nature of CRP as an innate immune pattern recognition molecule involved in the inflammatory cascade of autoimmune diseases remains unchanged (9). Meanwhile, as a key regulator of complement system activation, CRP expression level is closely correlated with core pathological processes in autoimmune diseases including complement activation and innate immune response. This is fully consistent with previous findings on the biological function of CRP in regulating complement activation via binding to immune complexes and inhibiting IFN-I production by plasmacytoid dendritic cells (pDCs (11, 33),. Our findings clarify the immunological basis of CRP as a shared core inflammatory molecule in autoimmune diseases at the omics level, and provide solid theoretical and experimental evidence for its application as a cross-disease broad-spectrum biomarker for initial inflammatory screening.

On the basis of the shared upregulation feature, this study further systematically elucidated the heterogeneity of CRP expression across different autoimmune diseases, as well as its corresponding disease-specific diagnostic and early warning value, providing precise evidence-based basis for its individualized clinical application. For the SLE cohort, we systematically dissected the expression characteristics and clinical significance of CRP in lupus nephritis (LN), a key complication of SLE, based on clinical data from a large cohort of 4534 patients. The results showed that although patients with LN had significantly higher erythrocyte sedimentation rate (ESR), Systemic Lupus Erythematosus Disease Activity Index 2000 (SLEDAI-2000) score, and heavier comorbidity burden than SLE patients without renal involvement, there was no statistically significant difference in circulating CRP levels between the two groups. Restricted cubic spline (RCS) models further confirmed that circulating CRP level had no significant independent association with the risk of LN onset, while ESR level was significantly positively correlated with LN onset risk. These findings directly verified the dissociation between CRP expression and renal organ involvement phenotype in SLE, which is the core clinical manifestation of the “CRP paradox”. Meanwhile, correlation analysis revealed that circulating CRP levels in SLE patients were still significantly positively correlated with ESR and SLEDAI-2000 scores, suggesting that CRP is not completely unrelated to SLE disease activity, but its indicative efficacy is interfered by the core pathological signals of the disease. In addition, CRP levels were significantly positively correlated with serum complement 3 (C3) and complement 4 (C4) levels, significantly negatively correlated with 24-hour urinary protein quantification, and had no significant correlation with immunoglobulin G (IgG) levels, further indicating that CRP is not only a biomarker of systemic inflammation in SLE, but its expression level is also closely related to complement system activation and the progression of renal immune injury. These results are highly consistent with decades of clinical research conclusions, and further improve the stratified application value of CRP in SLE. As early as 1981, Bravo et al. found that significant CRP elevation in SLE patients was mostly associated with complications such as infection, rather than simple lupus activity (34). Subsequent multiple meta-analyses also confirmed that CRP is a core biomarker for distinguishing infection from lupus flare in SLE (22). Our study further clarified the expression characteristics of CRP in different organ involvement phenotypes of SLE, especially verified its combined application value with ESR in LN risk assessment. This provides direct evidence-based basis for the combined application of inflammatory biomarkers to evaluate disease status and organ involvement risk in SLE, and addresses the core clinical pain point proposed in the introduction that reliable biomarkers are lacking for risk stratification of SLE complications (35).

For Sjögren’s Disease (SJD), the results of this study further clarified the clinical expression characteristics of CRP in pSS, providing new evidence for understanding its application value and the controversies in previous studies. The classic study by Moutsopoulos et al. in 1983 only found mild to moderate CRP elevation in 11 out of 50 pSS patients, and concluded that it was not related to clinical phenotypes (32). In contrast, our study based on clinical data from 102 pSS patients confirmed that serum CRP levels in pSS patients were significantly higher than those in healthy controls, suggesting that CRP can effectively reflect the systemic inflammatory burden accompanied by pSS. However, no statistically significant difference in circulating CRP and ESR levels was observed between pSS patients with interstitial lung disease (SS-ILD) and those without pulmonary involvement. This result provides a reasonable explanation for the controversies in previous studies: CRP elevation in pSS patients mainly reflects the systemic inflammatory burden, while for pulmonary involvement, an organ-specific complication, the indicative efficacy of circulating CRP is limited. This may be related to the characteristics of the local inflammatory microenvironment in SS-ILD, as well as the heterogeneity of local CRP synthesis and peripheral circulation release. It also differs from the findings of Lin et al. that ESR is significantly correlated with pSS-related ILD, suggesting that different inflammatory biomarkers have complementary value in the assessment of organ involvement in pSS (36). Meanwhile, in the pSS cohort included in this study, there was no significant difference in the baseline utilization rates of glucocorticoids and immunosuppressants between patients with and without ILD, which minimized the interference of treatment factors on inflammatory biomarkers and provided reliable cohort evidence for this conclusion. This also points out the direction for subsequent exploration of precise biomarkers for organ-specific complications of pSS, and the latest cohort studies have also listed CRP as an important reference index for the assessment of systemic inflammatory burden and long-term prognosis in pSS patients (37).

In the autoimmune cholangiopathy subgroup, we systematically dissected the disease-specific remodeling characteristics of CRP expression profile in the liver microenvironment of patients with primary sclerosing cholangitis (PSC) and primary biliary cholangitis (PBC) at single-cell resolution via liver single-cell RNA sequencing, filling the gap in previous studies on the cellular origin and spatial distribution of CRP in the local microenvironment of autoimmune liver diseases. Most previous studies focused on the correlation between circulating CRP levels and disease activity of autoimmune liver diseases, confirming that circulating CRP levels in PBC and PSC patients are closely related to liver inflammatory burden and disease severity, but the cellular origin and expression regulation characteristics of local CRP in the liver have not been clarified (38–40). Based on the integrated analysis of 189,938 high-quality liver single cells, our study systematically verified the cell type specificity and disease-specific remodeling of hepatic CRP expression under physiological and pathological conditions. Under physiological conditions, CRP in healthy liver only maintained an extremely low basal expression level in cholangiocyte and hepatocyte subpopulations, with no positive expression detected in all other intrahepatic stromal and immune cell subpopulations, presenting a high degree of cell type specificity. Under the pathological state of autoimmune cholangiopathies, both the expression level and expression scope of CRP in liver tissues underwent significant disease-specific remodeling. Specifically, in liver tissues from PSC patients, the overall expression level of CRP was significantly upregulated compared with healthy controls, with hepatocytes and biliary epithelial cells remaining the core cell types for CRP expression. Meanwhile, marked ectopic positive expression of CRP emerged in inflammation-related myeloid cell subpopulations including monocytes and activated macrophages, and scattered upregulation of CRP expression was also observed in liver sinusoidal endothelial cells and infiltrating lymphocytes. In liver tissues from PBC patients, the magnitude of CRP upregulation was even more pronounced. In addition to the substantial elevation of CRP expression in hepatocyte and biliary epithelial cell subpopulations, widespread positive CRP expression was detected in nearly all intrahepatic infiltrating immune cell subpopulations, including monocytes/macrophages, B lymphocytes, and T lymphocytes. Both the expression scope and expression intensity of CRP in the PBC group were significantly higher than those in the PSC group and healthy control group. This finding not only clarifies the disease-specific heterogeneity of CRP expression in autoimmune cholangiopathies, but also suggests that the chronic inflammatory and fibrotic microenvironment in the liver can induce ectopic CRP synthesis in multiple cell subpopulations. Furthermore, the locally ectopically expressed CRP is not merely an inflammatory biomarker, but may directly participate in the local inflammatory cascade and tissue injury process in the liver via a paracrine mode, providing a novel single-cell perspective for the research on the pathogenesis of autoimmune cholangiopathies.

To further verify the population extrapolation of our findings, this study systematically validated the expression characteristics and clinical correlation of high-sensitivity C-reactive protein (hs-CRP) in arthritis populations based on the nationally representative large-scale population data from the 2017–2018 cycle of the National Health and Nutrition Examination Survey (NHANES). Most previous studies on CRP in inflammatory arthritis were single-center small cohorts, which were difficult to exclude the interference of confounding factors such as demographic characteristics, lifestyle, and comorbidities. In contrast, this study strictly followed the official Guidelines for High Quality Analyses of NHANES Data issued by the National Center for Health Statistics (NCHS), and confirmed based on large-scale population data of 4497 participants that serum hs-CRP levels in patients with arthritis were significantly higher than those in healthy controls without arthritis. Subgroup analysis showed that although hs-CRP levels in patients with different arthritis subtypes including osteoarthritis (OA), RA, and PsA were all significantly higher than those in healthy controls, there was no statistically significant difference in hs-CRP levels among the subtypes. Meanwhile, correlation analysis revealed that serum hs-CRP levels were significantly positively correlated with peripheral blood neutrophil count and platelet count, and significantly negatively correlated with hemoglobin level, which directly verified that hs-CRP can effectively reflect systemic inflammatory burden and chronic inflammation-related anemia status in a nationally representative population. These results are fully consistent with the clinical practice of RA diagnosis and treatment: CRP has been incorporated into the EULAR/ACR-recommended criteria for RA risk stratification and remission assessment, and is a core index for the evaluation of RA disease activity (41, 42). Our study verified the broad-spectrum screening value of hs-CRP for arthritis in the national general population, providing high-level evidence-based medical evidence for its population-level clinical promotion in primary care settings. Beyond autoimmune and inflammatory diseases, elevated CRP levels are also a confirmed risk factor for cardiovascular disease, further highlighting the clinical significance of monitoring this inflammatory biomarker in diverse patient populations (1).

Based on the above findings, this study proposes a novel clinical application strategy of “broad-spectrum initial screening + disease-specific phenotype combined interpretation” for CRP, breaking through its century-old application limitation as a “non-specific inflammatory biomarker”. The core of this strategy is to conduct stratified clinical interpretation and application of CRP based on its shared expression characteristics and heterogeneity across different autoimmune diseases. In primary care settings, CRP testing can be used for broad-spectrum initial screening of systemic inflammatory status related to autoimmune diseases, to achieve early identification and specialist referral of high-risk populations. In specialist clinical settings, it is necessary to interpret CRP results individually in combination with disease type, specific clinical phenotypes, and other inflammatory biomarkers (such as ESR). For example, in SLE, attention should be paid to the dissociation between CRP and ESR to distinguish simple disease activity from complication risk; in autoimmune cholangiopathies, disease severity should be evaluated in combination with circulating CRP levels and local liver inflammatory characteristics; in arthritis, CRP levels can be used to assist in the assessment of systemic inflammatory burden and disease activity. This strategy fully utilizes the clinical advantages of CRP testing, which is simple, rapid, and highly accessible, while avoiding its limitation of non-specificity, and provides a consistent, easily accessible quantitative reference tool for the whole-process diagnosis and treatment of autoimmune diseases.

This study still has certain limitations. First, the clinical cohort of this study adopted a single-center cross-sectional design, which can only confirm the association between CRP levels and autoimmune diseases and their complications, but cannot clarify the causal relationship between them. Notably, this study focused exclusively on baseline CRP levels collected prior to pharmacotherapy or induction therapy and did not investigate the dynamic changes of CRP during the entire disease course (e.g., fluctuations with disease flares, remission, or long-term treatment response). More importantly, due to the lack of long-term follow-up data, we are unable to elucidate the long-term predictive value of baseline CRP levels for future adverse outcomes such as cardiovascular events in patients with autoimmune diseases, despite CRP being a well-established risk factor for cardiovascular diseases. This cross-sectional design thus also limits our ability to explore the longitudinal correlation between baseline CRP levels and cardiovascular risk stratification in this patient population. The cross-sectional design thus limits our ability to clarify how CRP levels evolve with disease progression, and in the future, multicenter prospective cohort studies are needed to further verify the long-term predictive value of CRP for the onset risk of autoimmune diseases and the occurrence of complications, the occurrence of complications and cardiovascular events, as well as to track the dynamic changes of CRP throughout the disease course. Second, this study did not conduct in-depth analysis on the expression differences between pentameric CRP (pCRP) and monomeric CRP (mCRP) in different autoimmune diseases. Previous studies have confirmed that CRP of the two conformations has completely different biological functions, mCRP has stronger pro-inflammatory activity, and anti-mCRP autoantibodies are closely related to the disease activity of SLE and pSS (14, 19). In the future, conformation-specific CRP detection should be improved to more accurately analyze its functional differences and clinical value in different autoimmune diseases. In addition, the single-cell transcriptomic analysis in this study was based on public datasets, and no clinical samples from our center were included for synchronous verification. In the future, it is necessary to expand the sample size of human primary tissue samples, and further verify the specific pathogenic mechanism of locally ectopically synthesized CRP in inflammatory injury and fibrosis of autoimmune cholangiopathies via *in vivo* and *in vitro* functional experiments, to clarify its value as a potential therapeutic target.

In conclusion, through multi-dimensional integrated analysis of single-center clinical cohort, pan-disease panoramic proteomics, liver single-cell RNA sequencing, and nationally representative large-scale population data from NHANES, this study comprehensively mapped the expression landscape of CRP in multiple autoimmune diseases, and elucidated its cross-disease shared expression characteristics, inter-disease expression heterogeneity, cellular origin at single-cell resolution, and clinical application value. The findings not only provide a novel perspective for the research on the innate immune biology of CRP, but also establish a precise stratification and inflammation assessment system for autoimmune diseases based on CRP, providing a simple, reliable, and scalable laboratory tool for clinical diagnosis and treatment. In the future, further exploration of the conformation-specific pathogenic mechanism and targeted therapeutic strategy of CRP based on the findings of this study will open up a new direction for the precise diagnosis and treatment of autoimmune diseases.

## Data Availability

The SomaScan proteomics dataset, as well as the GSE201006, GSE245620, GSE125188, and GSE115469 datasets used in this study, are publicly available datasets from the GEO and SomaLogic platforms and can be accessed free of charge from the respective platforms. The NHANES 2017-2018 data are publicly available for download from the official website of the U.S. Centers for Disease Control and Prevention (CDC, https://wwwn.cdc.gov/nchs/nhanes/).
